# The Bacterial G_q_ Signal Transduction Inhibitor FR900359 Impairs Soil-Associated Nematodes

**DOI:** 10.1007/s10886-023-01442-1

**Published:** 2023-07-15

**Authors:** Wiebke Hanke, Judith Alenfelder, Jun Liu, Philipp Gutbrod, Stefan Kehraus, Max Crüsemann, Peter Dörmann, Evi Kostenis, Monika Scholz, Gabriele M. König

**Affiliations:** 1 Institute for Pharmaceutical Biology, University of Bonn, Nussallee 6, D-53115 Bonn, Germany; 2 Molecular, Cellular and Pharmacobiology Section, Institute for Pharmaceutical Biology, University of Bonn, Nussallee 6, D-53115 Bonn, Germany; 3 Neural Information Flow, Max Planck Institute for Neurobiology of Behavior – CAESAR, Ludwig-Erhard-Allee 2, D-53175 Bonn, Germany; 4 Institute of Molecular Physiology and Biotechnology of Plants (IMBIO), University of Bonn, Karlrobert-Kreiten-Straße 13, D-53115 Bonn, Germany; 5 Present Address: Bonn International Graduate School – Land and Food, University of Bonn, Katzenburgweg 9, D-53115 Bonn, Germany

**Keywords:** FR900359, *Chromobacterium vaccinii*, Soil microbiome, Nematodes, *Heterodera schachtii*, *Caenorhabditis elegans*

## Abstract

The cyclic depsipeptide FR900359 (FR) is derived from the soil bacterium *Chromobacterium vaccinii* and known to bind G_q_ proteins of mammals and insects, thereby abolishing the signal transduction of their G_q_ protein-coupled receptors, a process that leads to severe physiological consequences. Due to their highly conserved structure, G_q_ family of proteins are a superior ecological target for FR producing organisms, resulting in a defense towards a broad range of harmful organisms. Here, we focus on the question whether bacteria like *C. vaccinii* are important factors in soil in that their secondary metabolites impair, e.g., plant harming organisms like nematodes. We prove that the G_q_ inhibitor FR is produced under soil-like conditions. Furthermore, FR inhibits heterologously expressed Gα_q_ proteins of the nematodes *Caenorhabditis elegans* and *Heterodera schachtii* in the micromolar range. Additionally, *in vivo* experiments with *C. elegans* and the plant parasitic cyst nematode *H. schachtii* demonstrated that FR reduces locomotion of *C. elegans* and *H. schachtii*. Finally, egg-laying of *C. elegans* and hatching of juvenile stage 2 of *H. schachtii* from its cysts is inhibited by FR, suggesting that FR might reduce nematode dispersion and proliferation. This study supports the idea that *C. vaccinii* and its excreted metabolome in the soil might contribute to an ecological equilibrium, maintaining and establishing the successful growth of plants.

**Supplementary Information:**

The online version contains supplementary material available at 10.1007/s10886-023-01442-1.

## Introduction

Nematodes are ubiquitously distributed in soil (Bardgett and van der Putten 2014; van den Hoogen et al. 2019) and present a widespread phylum with huge diversity (Porazinska et al. 2009; Song et al. 2017; Kouser et al. 2021; Lazarova et al. 2021). Their functional role in soil has been investigated intensively due to their impact on the soil food web and soil health (Ingham et al. 1985; Procter 1990; Ferris 2010; Lazarova et al. 2021; Melakeberhan et al. 2021). Nematodes are divided into trophic groups depending on their food source, i.e., bacterivore, fungivore, omnivore, herbivore, and predator (Yeates et al. 1993; van den Hoogen et al. 2019). Herbivorous nematodes, i.e., plant-parasitic nematodes, are known plant pathogens causing 12.3% of crop losses, equal to 157 billion US Dollar annually (Singh et al. 2015). The other trophic groups are known beneficial nematodes (Trap et al. 2016), e.g., predators like entomopathogenic nematodes (Koppenhöfer et al. 2020) are lethal for insect pests and utilized as biocontrol agents in agriculture (Dillman and Sternberg 2012; Kenney and Eleftherianos 2016). To sustain plant health, it is important to understand the equilibrium of beneficial and pathogenic effects present in soil, e.g., through investigations of interactions of soil organisms and their excreted metabolites.

**Fig. 1:**
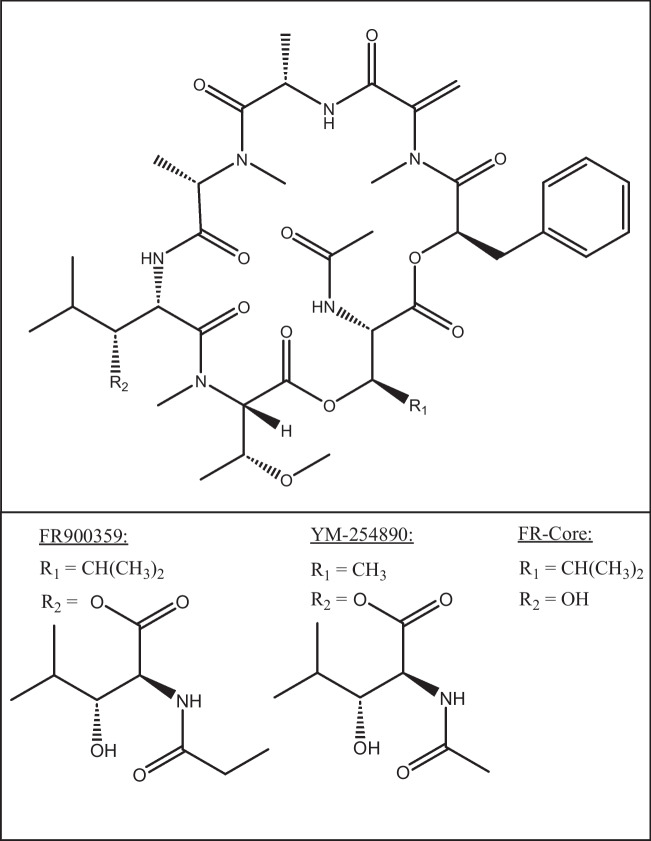
Structures of FR900359, YM-254890, and FR-Core

The cyclodepsipeptide FR900359 (FR) (Fig. 1) is a member of a small family of natural products known as chromodepsins (Hermes et al. 2021a). The monomeric side chain of FR, *N*-propionylhydroxyleucine (*N*-Pp-OH-Leu), is attached via an ester bond to a hydroxyl group of one of the OH-Leu units of the macrocyclic part of the molecule. FR is produced by “*Candidatus* Burkholderia crenata”, an endosymbiotic nonculturable bacterium with a highly reduced genome living in the leaf nodules of the higher plant *Ardisia crenata* (Fujioka et al. 1988; Miyamae et al. 1989; Crüsemann et al. 2018). Biosynthesis of FR is performed by two nonribosomal peptide synthetase systems, encoded by the biosynthetic gene cluster (BGC) *frs* consisting of *frsA*-*H* (Crüsemann et al. 2018; Hermes et al. 2021b). After discovery of the *frs* BGC, database sequence searches allowed us to identify the cultivable and free-living soil bacterium *Chromobacterium vaccinii* as an additional FR producer (Hermes et al. 2021b). Other structurally related chromodepsins, i.e. FR-3 (sameuramide), were also found in an ascidian of the family *Didemnidae* (Yamashita et al. 2011), and another FR congener termed YM-254890 (YM) (Fig. 1) and its derivatives were reported from the soil bacterium *Chromobacterium* sp. QS3666, isolated in Japan (Taniguchi et al. 2003a). The structure of YM (Fig. 1) is almost identical to that of FR, differing only at two positions, (i) the side chain is composed of *N*-acetylhydroxyleucine (i.e. an acetyl instead of a propionyl moiety), and (ii) instead of the *N*-acetylhydroxyleucine unit in FR, YM possesses a *N*-acetylthreonine residue in the macrocyclic part (Fig. 1) (Taniguchi et al. 2003b).

FR and YM interact with Gα_q_ proteins and thereby inhibit G_q_-mediated nucleotide exchange with high selectivity at micromolar potency (Schrage et al. 2015). YM and FR differ in their residence time, but not in their binding affinity or inhibitory potency towards Gα_q_ (Kuschak et al. 2019; Voss et al. 2021). This makes them both extremely useful tools to study G_q_-mediated signaling of G protein-coupled receptors (GPCR) (Kamato et al. 2017; Kostenis et al. 2020). The latter are responsible for many major physiological processes and the target of approx. 35% of our pharmaceutical drugs (Insel et al. 2019). Interestingly, there are only four major Gα protein families, Gα_q_ being one of them (Downes and Gautam 1999). G_q_ is hence part of many of the over 800 different GPCR-dependent signaling pathways. Thus, with chromodepsins like FR and YM, it is possible to modulate with a single active substance (e.g., FR or YM) the response of many GPCRs. This may be highly relevant for both the medical use and the ecological impact of FR and YM. Medically important findings include possible treatment of airway disorders (Carr et al. 2016; Matthey et al. 2017), reduction of adipositas (Klepac et al. 2016), and suppression of uveal melanoma (Annala et al. 2019; Lapadula et al. 2019; Onken et al. 2022), whereas the ecological effect of FR has mainly been researched towards insects (Crüsemann et al. 2018).

Indeed, as previous experiments towards insects and mammals showed, FR may function in nature as a protectant for the bacterial producer and, as in the case of endosymbiotic bacteria, its host plants. FR displays strong affinity towards Sf9 insect cell membranes and G_q_ proteins of the *Bombyx mori* and *Bemisia tabaci*. A second experiment using nymphs of the beetle *Riptortus pedestris* measured their survival rate after exposure to different FR concentrations over 9 days. For the two highest FR concentrations, 40 µM and 200 µM, the survival rate started to decline drastically after 4 days, with all insects being dead after seven days (Crüsemann et al. 2018). FR activity was also tested in mice (Matthey et al. 2017) and rats (Miyamae et al. 1989) to investigate its effects on mammals. Intratracheal application of FR in mice lead to airway relaxation, which may be of medical importance (Matthey et al. 2017), but systemic oral application in mice and rats resulted in decreased blood pressure and transient bradycardia (Miyamae et al. 1989; Matthey et al. 2017; Meleka et al. 2019).

Given its selective and potent activity, it can be assumed that the structures of FR and YM were evolutionary optimized for G_q_ inhibition. Structural changes in most positions of the backbone and side chain caused a drastic loss of activity (Xiong et al. 2016, 2019; Zhang et al. 2018), e.g., FR-Core (Fig. 1), a FR molecule without the *N*-propionylhydroxyleucine side chain is 13-fold less active in dynamic mass redistribution (DMR) assays than FR and shows 207-fold lower binding affinity to its target than FR itself. It was thus hypothesized, that FR-Core is the evolutionary ancestor molecule of FR and, considering G_q_ inhibition as a trait for positive selection, that side chain biosynthesis evolved by duplications of sequences within *frs* (Hermes et al. 2021b).

The FR producing bacterium *C. vaccinii* MWU205 has originally been isolated from wild cranberry bog soil (Soby et al. 2013). If this bacterial strain indeed produces FR *in situ*, it could protect plants growing in bacteria-containing soil from pathogens like plant pathogenic nematodes. To date, *C. vaccinii* was only cultivated for FR production under laboratory conditions and it is not clear, whether chromodepsins like FR are produced in soil. We therefore started experiments to investigate whether *C. vaccinii* is able produce FR under soil-like conditions, and if FR is secreted by the producer bacterium into its surrounding. Next, we used our knowledge regarding the FR-binding site to approximate sensitivity of G_q_ proteins of nematodes to FR *in silico*, which was subsequently substantiated by *in vitro* assays using heterologously expressed nematode Gα_q_. Most importantly, ecological relevant effects of FR on the soil-associated nematodes model organism *Caenorhabditis elegans* and *Heterodera schachtii*, a plant parasitic nematode, were observed *in vivo*. In these experiments FR affected locomotion and egg-laying of *C. elegans*. Similarly, experiments with *H. schachtii* resulted in inhibition of locomotion and hatching of juvenile stage 2 (J2) nematodes by FR.

Our data suggest that bacteria like *C. vaccinii* are important members of the soil microbiome, as their biosynthetic products may contribute to an ecological equilibrium and may even serve to protect plants from detrimental nematodes.

## Methods and Materials

### General Experimental Procedures

Nuclear magnetic resonance (NMR) spectra were recorded on a Bruker Ascend 600 NMR spectrometer operating at 600 MHz (^1^H) and 150 MHz (^13^C) using CDCl_3_ as solvent (Deutero GmbH; 99.8% D). NMR spectra were processed using Bruker Topspin Version 1.3 or MestReNova 8.0.1 software. Spectra were referenced to residual solvent signals with resonances at δ_H__/__C_ 7.26/77.0*.* High-performance liquid chromatography mass spectrometry (HPLC/MS) data were recorded on a Waters 2695 separation module, which was coupled to a Waters 996 photodiode array detector, and a Waters QDa detector with electrospray ionization source. For separation a gradient elution with mobile phases A (acetonitrile/water 5/95 with 5 mM ammonium acetate and 40 µL acetic acid per Liter) and B (acetonitrile/water 95/5 with 5 mM ammonium acetate and 40 µL acetic acid per liter) on a Waters X Bridge Shield RP_18_ column (100 × 2.1 mm; 3.5 μm) at 25 °C were used (flow of 0.3 mL/min, 80/20 A/B to 0/100 A/B within 20 min, and hold for 10 min). MS data were collected in positive and negative mode in the range between *m/z* 140–1250 and additionally in the positive single ion mode for the mass trace of FR (*m/z* 1002.5; M + H^+^). HPLC was carried out either using a Waters HPLC system, controlled by Waters Millenium software, consisting of a 600E pump, a 996 PDA detector, and a 717 plus autosampler or on a Waters Breeze HPLC system equipped with a 1525µ dual pump, a 2998 photodiode array detector, and a Rheodyne 7725i injection system.

### Sample and Organism Collection

*C. vaccinii* MWU205 (DSM 25150, ATCC BAA 2314) was purchased from the German Collection of Microorganisms and Cell Cultures, DSMZ. *H. schachtii* was kindly provided by Dr Philipp Gutbrod. *C. elegans* strains N2, *egl-30(n686)*, *egl-30(ad806)*, *egl-30(ad805)*, *dgk-1(sy428)*, *eat-16(sa609)*, and *Escherichia coli* strain OP50 were provided by the Caenorhabditis Genetics Center, which is funded by NIH Office of Research Infrastructure Programs (P40 OD010440)*. C. elegans* was maintained at 20 °C using standard methods.

Topsoil was sampled in a garden on the 2^nd^ of April 2019 (Dortmund Eichlinghofen, North Rhine-Westphalia: 51°28′35.0 “N 7°24′22.1 “E) and dried at room temperature for seven days on paper. Soil was sieved to remove rocks or plant debris and stored at 4 °C.

### Cultivation and Extraction of *C. vaccinii*

Lysogeny broth (LB) medium was prepared using 10 g/L NaCl, 10 g/L tryptone, 5 g/L yeast extract. A LB agar plate was inoculated with a cryoculture, cultivated for 2–4 days at 25 °C, and used afterwards to inoculate 20 mL LB medium for preculture. The preculture was grown for 24 h at 25 °C and 180 rpm. Subsequently the main culture (1.5 L LB medium) was cultivated with 1% of the preculture and 1‰ of carbenicillin (final concentration of 50 µg/mL) at 25 °C and 160 rpm for 36–48 h. Extraction was performed with *n*-butanol (1:1) overnight, followed by centrifugation at 4,000 rpm for 10 min. The upper phase was collected and evaporated.

### Isolation of FR and FR-Core

Each purification step was accompanied by MS analysis of the fractions. The crude material was fractionated on a Reveleris C_18_ flash column (220 g, 40 µm). A stepwise gradient solvent system of increasing polarity and a flow rate of 65 mL/min was used starting with 50/50 H_2_O/MeOH for 13 min, then changing to 30/70 H_2_O/MeOH within 1 min and hold again for 13 min. The gradient was changed then within 1 min to 25/75 H_2_O/MeOH and hold for 25 min, then within 1 min to 20/80 H_2_O/MeOH, hold for 13 min, then within 1 min to 15/85 H_2_O/MeOH and hold for 25 min. Finally, the gradient was changed within 1 min to 100% MeOH and hold for additional 10 min. According to the measured evaporative light scattering detector (ELSD) and UV signals, a FR containing fraction was collected at 70 min. Final purification was done by HPLC with a semi-preparative Macherey–Nagel Nucleodur C_18_ column (250 × 8 mm, 5 µm) using an isocratic elution with 20/80 H_2_O/MeOH (flow 2.0 mL/min). Pure FR was isolated as a white powder (FR: t_R_: 20 min). The identity and purity were confirmed using NMR (Supplementary Figures S11-S12). FR-Core was isolated as described elsewhere (Hermes et al. 2021b).

### FR Calibration Curve

Eight different concentrations of FR (purity > 90%) in HPLC/MS grade methanol (0, 0.0001, 0.0005, 0.001, 0.005, 0.01, 0.05, 0.1 mM FR) were prepared for HPLC/MS analyses. For small concentrations, calibration curves were calculated using the four smallest concentrations. Visualization was performed using Prism 9.5.0.

### Cultivation of *C. vaccinii* in Soil-Extracted Medium

Soil was extracted using the soil-extracted solubilized organic matter (SESOM) protocol (Vilain et al. 2006). Therefore 100 mg of soil were mixed with 0.5 L 3-(*N*-morpholino) propanesulfonic acid (MOPS) buffer (1.05 g MOPS, 0.09 g disodium EDTA, and 0.10 g sodium acetate were solved in water, and pH was adjusted to 7 at 40 °C) and shaken at 160 rpm for two hours. Afterwards the whole mixture was filtered multiple times (folded filter paper for qualitative work, then vacuum filtration using 5 µm and subsequently 0.45 µm polyvinylidene fluoride filters). The pH of the extract was adjusted to 7.1–7.3 and the whole extract was subsequently sterile filtered (0.2 µm polyether sulfone membrane). For chitin experiments, chitin was autoclaved directly in the bottle used for sterile filtration of the final SESOM (final chitin concentration: 1 g/L) now called SESOM ( +). Both SESOM and SESOM ( +) were tested for sterility by inoculation of a LB plate and incubating it at 37 °C for 24 h.

Precultures for SESOM experiments were grown in LB medium inoculated with *C. vaccinii* grown on LB plates for 2–4 days at 25 °C. The preculture was supplemented with carbenicillin disodium salt to a final concentration of 50 µg/mL. After cultivation at 25 °C for 24 h the preculture was centrifuged at 5.000 rpm for 5 min. The pellet was washed two times with 0.9% NaCl solution. Afterwards the colony-forming units (CFU) were determined by plate count and inoculation was performed with 3.2 × 10^4^ CFU/µL for SESOM and 5.2 × 10^6^ CFU/µL for SESOM ( +). For SESOM 2 × 80 mL were inoculated. One culture was separated at the start (0 days) into three samples à 25 mL, which were directly extracted. The second culture was separated similarly after 5 days of cultivation at 25 °C and 180 rpm. One blank was prepared using only SESOM. Extraction (1:1) was performed for 6 h at 180 rpm using *n*-butanol. Afterwards the whole extract was centrifuged for 15 min at 4,000 rpm and the upper phase was evaporated. For analysis via HPLC/MS 1 mg/mL solutions were prepared using HPLC/MS grade methanol. For SESOM ( +), eight 50 mL flasks containing 30 mg chitin were sterilized and afterwards filled with SESOM. One flask was not inoculated and directly extracted as blank. Seven flasks were inoculated and three of these flasks were extracted (0 days). Four flasks were cultivated at 25 °C and 180 rpm and extracted after five days. Extraction (1:1) was performed over night at 180 rpm using *n*-butanol. Subsequently the whole extract was centrifuged for 15 min at 4,000 rpm and the upper phase was evaporated. For analysis via HPLC/MS, 2 mg/mL solutions were prepared using HPLC/MS grade methanol.

### FR Secretion Experiment

The preculture was made as described in “*Cultivation and Extraction of C. vaccinii*”. Main cultures (6 times) were prepared using 25 mL LB medium inoculated with the preculture (1%). The preculture and main cultures were supplemented with carbenicillin disodium salt to a final concentration of 50 µg/mL. After 43 h the main culture was centrifuged at 5,000 rpm for 5 min, the pellet was washed two times with 2.5 mL 0.9% NaCl-solution and centrifuged again. All three supernatants were combined and extracted with 30 mL *n*-butanol. Additionally, the pellet was extracted with *n*-butanol, sonicated shortly, and shaken over night at 180 rpm. After centrifugation at 4,000 rpm for 10 min, the *n*-butanol phase was evaporated. All extracts (supernatant and pellet) were weight and solved for HPLC/MS with HPLC/MS grade methanol to a concentration of 2 mg/mL.

### Bioinformatical Alignment and Visualization

The Basic Local Alignment Search Tool (BLAST) (Altschul et al. 1997, 2005) was utilized using the amino acid sequence of the Gα_q_ isoform a of *C. elegans* (UniProt: G5EGU1) as query. Standard databases (non-redundant protein sequences) were selected as search set but restricted to the organisms belonging to Nematoda. The blastp algorithm was used with default parameters.

Sequences were aligned using the Clustal W alignment tool in MEGA 11 (Version 11.0.11). For the pairwise alignment, a penalty of 10 for gap opening and 0.1 for gap extension were selected. For the multiple alignment, the penalty for gap opening was the same, but gap extension was punished with 0.2. Gonnet was chosen as protein weight matrix, and residue-specific and hydrophilic penalties were switched on. Concerning gap separation, a matrix of four and no end were selected. No negative matrix was used, and the delay divergent cutoff was set at 30%.

Depictions of YM in complex with a chimeric G_i1/q_ protein were created with PyMOL™ 2.5.4 (Schrodinger) from PDB ID 3AH8 (Nishimura et al. 2010).

### Cell Culture and Transient Transfection

Cell culture materials were purchased from Invitrogen. The Gα_q/11_ knock-out HEK293 cells (HEK-∆G_q/11_) were generated by CRISPR-Cas9 technology as described previously in detail (Schrage et al. 2015). Cells were cultivated in Dulbecco’s modified Eagle’s medium, supplemented with 10% (v/v) fetal calf serum, penicillin (100 U/mL) and streptomycin (0.1 mg/mL), at 5% CO_2_ and 37 °C in a humidified atmosphere. All monthly tests for mycoplasma contamination by PCR were negative.

HEK-∆G_q/11_ cells were transfected in suspension 48 h prior to the experiments using polyethylenimine (1 mg/mL, Polyscience) following the manufacturer’s protocol. A total amount of 8 µg plasmid (3 µg, 0.6 µg, 2 µg of expression plasmids containing HA-tagged Gα_q_ isoforms, muscarinic acetylcholine M3 receptor, and RIC-8A, respectively, filled up with pcDNA3.1( +)) and 24 µL PEI solution were added to 2.8 × 106 cells plated in 10 cm dishes.

### IP_1_ Accumulation Assay

The IP_1_ accumulation was measured using homogeneous time resolved fluorescence (HTRF) technology (Cisbio) following the manufacturer’s instructions. For this, transfected HEK-∆G_q/11_ cells were detached and washed in PBS. After resuspension in LiCl-containing assay buffer stopping breakdown of IP_1_, cells were seeded into white 384-well plates with 50,000 cells per well. Carbachol and FR were added simultaneously, followed by 40 min of incubation at 37 °C. Subsequently, cells were lysed and incubated with d2-labeled and cryptate-labeled IP_1_ antibodies for a minimum of 60 min at room temperature. The HTRF ratio values measured with a Mithras LB 940 multimode plate reader (Berthold Technologies) were converted to IP_1_ concentrations in nM using an IP_1_ (unlabeled) standard curve.

### Calcium^2+^ Mobilization Measurement

Calcium^2+^ mobilization was measured using FLIPR Calcium 5 assay kit using the Flex Station 3 MultiMode Benchtop reader (both Molecular Devices), as described elsewhere (Patt et al. 2021) with slight modifications. Briefly, 24 h after transfection, HEK-∆G_q/11_ cells transferred to flat-bottom 96-well cell culture plates at a density of 60,000 cells per well. On the day of the assay, cells were incubated in Calcium 5 dye for 45 min at 37 °C before 1 to 3 dilutions with Hanks' Balanced Salt Solution + 20 mM HEPES. For preincubation with inhibitor, FR was added to the dye in the appropriate concentrations. Kinetic fluorescence measurements (baseline read and addition of agonist or buffer after 20 s) were performed to assess calcium^2+^ release from intracellular stores.

### *C. elegans* Synchronization

The investigated pathway directly impacts developmental timings of the worm. To compare animals at the same age, we measured the developmental time from egg to adulthood for all strains. We found that mutant worms at 20 °C needed 6 h more to reach adulthood than wildtype. For the locomotion experiments, the animal culture times were offset to allow delayed mutants to develop until the correct age. As checks for egg-laying during the locomotion experiments revealed the suppressor mutants, *dgk-1*(*sy428*), and *eat-16*(*sa609*), to be slower their delay was lengthened to 10 h for the subsequent egg-laying experiments. Synchronization of *C. elegans* wildtype N2, *egl-30*(*ad806*), *egl-30*(*ad805*), *egl-30*(*n686*), *dgk-1*(*sy428*), and *eat-16*(*sa609*) was performed using bleaching solution (2 mL 5% sodium hypochlorite, 1.5 mL 5 M potassium hydroxide, and 6.5 mL ddH_2_O), M9 buffer and *C. elegans* grown for 3–4 days on a 10 cm NGM plate at 20 °C. Nematodes were washed off with M9. After centrifugation and removal of 900 µL, 1 mL of bleaching solution was added to the pellet. The sample was mixed for 2 min and centrifuged afterwards. This step was repeated a second time. Afterwards the pellet was washed three times with M9 to remove the bleach solution and resuspended in 1 mL of M9 buffer. Finally, eggs were counted, and concentration was adjusted to a maximum of 5 eggs/µL, if necessary, and rotated overnight to synchronize to larval stage 1 (L1).

### *C. elegans* Tracking Experiment

Approximately 100 synchronized *C. elegans* N2, *egl-30*(*ad805*), *egl-30*(*n686*), *egl-30*(*ad806*), *dgk-1*(*sy428*), *eat-16*(*sa609*) L1 were grown on NGM with a spot (40 µL) *E. coli* OP50 mixed with FR (2.5 mM) in 1% DMSO or just 1% DMSO as control. After approx. 55 h (N2) plus above-mentioned offset for mutant worms were imaged. Imaging of worms was performed using a commercial upright epifluorescence microscope (Axio Zoom V16; Zeiss) equipped with a 1x objective (PlanNeoFluar Z 1.0x/N.A. 0.25). Brightfield image was performed and imaged on camera (BASLER; acA3088-57um) using a camera adapter with an additional 0.5 × magnification (60N-C ${~}^{2}\!\left/ \!{~}_{3}\right.$ 0.5x; Zeiss) resulting into an effective magnification on camera of 0.35x. Animals were imaged at 15 fps for 5 min unless otherwise indicated.

Animals were tracked using the tracking package trackpy (Allan et al. 2019) with a custom detection script in Python based on the pharaglow package (Bonnard et al. 2022). The animal speed was calculated from the resulting center-of-mass coordinates as follows: The trajectories (x, y, t) were sub-sampled from 14 fps to 2.8 fps and the speed was calculated as $v\left({t}_{2}\right)=\sqrt{\frac{{({x}_{t2}-{x}_{t1})}^{2}+{({y}_{t2}-{y}_{t1})}^{2}}{dt}}$ with $dt=\frac{1}{2.8 }s.$

The animals’ spatial distribution was evaluated by counting nematodes inside the lawn and outside the lawn, where outside the lawn meant that no part of the worm touched the lawn. Border crossings were counted in a similar way as the recorded tracks were evaluated and each crossing independent of the direction (leaving or entering) was counted.

### *C. elegans* Egg-Laying Experiment

Approx. 50 synchronized *C. elegans* N2, *egl-30*(*ad806*), *egl-30*(*n686*), *dgk-1*(*sy428*), *eat-16*(*sa609*) L1 were grown on NGM plates completely covered with *E. coli* OP50 mixed with FR (2.5 mg/mL) in 1% DMSO or 1% DMSO as control for 72 h (N2), 78 h (*egl-30*(*n686*), *egl-30*(*ad806*)), and 81 h (*dgk-1*(*sy428*), *eat-16*(*sa609*)). Afterwards 12 worms per genotype were picked and put separately on plates completely covered with the mixtures described before. After two hours the nematode was removed from the plate and eggs were counted.

### *C. elegans* Retained Eggs Experiment

Approx. 100 synchronized *C. elegans* N2, *dgk-1*(*sy428*), *eat-16*(*sa609*) L1 were grown on NGM plates completely covered as described for the egg-laying experiment. After 72 h (N2) and 81 h (mutants), 20 worms were picked and put separately in 10 µL drops of 6% bleaching solution (5% sodium hypochlorite solution solved in dH_2_O) for 15 min.

### *H. schachtii* Cultivation

*H. schachtii* was cocultivated with *Sinapis alba* to generate cysts and J2 used for the activity and cyst assay (Sijmons et al. 1991).

### *H. schachtii* Activity Assay

Approximately 100 J2 were added to 500 µL volume containing 1% DMSO mixed with or without 1 mM FR. Additionally one experiment was conducted by adding 50 µL octopamine to control and FR. After 4 d the number of active J2 was detemined by microscopic observation of their shape (active: coiled or wavy-like, inactive: straight or slightly curved line). For the concentration-dependency experiment different concentration of FR (0.0624 mM, 0.125 mM, 0.25 mM, 0.5 mM) were added and counted after 4 d (Gutbrod et al. 2020).

### *H. schachtii* Cyst Assay

Approximately 20 cysts were added to 500 µL volume containing 1% DMSO mixed with or without 0.01 mg/mL FR. Each experiment was replicated 4 times and cultivated for 7 d at room temperature. Afterwards the number of J2 was counted per experiment and divided by the number of cysts in the well.

### Statistical Analyses

Data and statistical analyses were performed using GraphPad Prism version 9.5.0 as described in detail below. To evaluate the spatial distribution of *C. elegans* a *modified two sample binomial test* (Wong et al. 2014) was performed using Microsoft Excel. Raw data for all statistical tests performed is summarized in Online Resource 2.

SESOM, SESOM with chitin, and the secretion experiment were tested for normality using the *Shapiro–Wilk test*, which stated them to be normally distributed. Afterwards the *unpaired t-test* for both SESOM experiments and the *paired t-test* for the secretion experiment were utilized. Summarized data represent the data of three technical replicates for SESOM, three or four replicated for SESOM with chitin, and six replicates for the secretion experiment. For the FR calibration curve for SESOM the four smallest concentrations were picked and for the secretion experiment all eight concentrations were chosen and used to calculate a simple linear regression using Prism 9.5.0.

*C. elegans* experiments concerning velocity, egg-laying rate, and retained eggs were tested for normality or lognormality using *D’Agostino & Pearson test*. For velocity all three *egl-30* mutants, *egl-30*(*ad805*), *egl-30*(*n686*), and *egl-30*(*ad806*) were found to have a log-normal distribution while N2, *dgk-1*(*sy428*), and *eat-16*(*sa609*) were not normally distributed. Subsequently, the *Mann–Whitney test* was performed for N2, *dgk-1*(*sy428*), and *eat-16*(*sa609*) and the *unpaired t-test* was performed for all three *egl-30* mutants to compare control against FR. For the egg-laying rate, all five genotypes (N2, *egl-30*(*n686*), *egl-30*(*ad806*), *dgk-1*(*sy428*), and *eat-16*(*sa609*)) were found to be normally distributed. Subsequently the *unpaired t-test* was chosen for the comparison of Control versus FR. For the retained eggs assay, *C. elegans* N2 and *eat-16*(*sa609*) displayed normally distributed data, while *dgk-1*(*sy428*) was not normally distributed. Accordingly, *unpaired t-tests* were performed to compare Control vs. FR for *C. elegans* N2 and *eat-16*(*sa609*), while the *Mann–Whitney test* was used for *dgk-1*(*sy428*).

Regarding *H. schachtii*, experiments evaluating the effect of FR and octopamine on the activity of nematodes, *One-Way-ANOVA* with *Tukey’s multiple comparisons test* was performed as data passed the *Shapiro–Wilk test* for normality. To investigate the effect of FR on hatching of J2 *H. schachtii*, the *unpaired t-test* was performed as data passed the *Shapiro–Wilk test* for normality. Summarized data represent the data of four technical replicates.

## Results

### Production and Excretion of FR by *C. vaccinii* MWU205 Under Soil-like Conditions

The functional assessment of single members of a microbiome is extremely difficult. Apart from the fact that only a small percentage of soil bacteria can be cultivated in the laboratory (van Pham and Kim 2012; Fierer 2017), their interactions and their secondary metabolite production under natural conditions are mostly unknown (Berendsen et al. 2013). Additionally, the *in situ* relevance of natural products is often not clear. To date we failed in cultivating *C. vaccinii* in the laboratory in soil directly. Even though this bacterium was shown to produce FR and derivatives under standard laboratory conditions (Hermes et al. 2021b), it is not clear whether secondary metabolite production also occurs under natural or close to natural conditions.

**Fig. 2:**
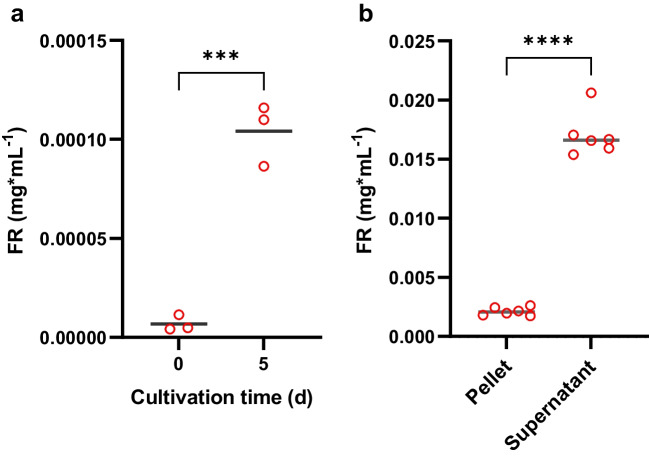
**a** FR concentration as determined after *n*-butanol extraction of a *C. vaccinii* culture in SESOM, cultivated for 5 days or extracted after inoculation (0 days). FR concentration was evaluated using HPLC/MS. For both experiments, *n* = 3. The significance was determined using a *two-tailed unpaired t-test*. **b** Concentration of FR (mg/mL) in supernatant and pellet of *C. vaccinii* liquid culture. *C. vaccinii* was cultivated for 43 h in LB medium and centrifuged afterwards, resulting in pellet and supernatant, which were extracted separately with *n*-butanol. Six repeats were performed. The significance was determined using a *paired t-test*. *P* > 0.05 = ns, *P* < 0.05 = *, *P* < 0.01 = **, *P* < 0.001 = ***, *P* < 0.0001 = ****

To address whether FR is produced and secreted by *C. vaccinii* under soil-like conditions, we decided to use soil extracts, particularly soil-extracted solubilized organic matter (SESOM), as a liquid medium. SESOM applies 3-(*N*-morpholino) propanesulfonic acid (MOPS) buffer for soil extraction. The resulting extract can be used for bacterial cultivations (Vilain et al. 2006). We collected soil samples, extracted SESOM, and applied it to cultivate *C. vaccinii* for 5 days at 25 °C. In a second experiment we added the natural polymer chitin to SESOM to supplement a potential carbon and nitrogen source from soil, as genes encoding for chitinases have been reported in the genome of *C. vaccinii* (Vöing et al. 2015). Extraction with *n*-butanol was done after inoculation (0 days) and after 5 days of cultivation, followed by analysis of the extracts via HPLC/MS, calculation of FR concentrations via calibration curves (Supplementary Fig. S1 of Online Resource 1), and comparison of the FR concentrations of the different experiments. Both experiments (i.e., with and without chitin) presented a significantly increased FR concentration after five days of cultivation, 15-fold for SESOM (*two-tailed unpaired t-test*: *p* = 0.0005) (Fig. 2a) and twofold (*two-tailed unpaired t-test*: *p* = 0.0121) for SESOM with chitin (Supplementary Fig. S2 of Online Resource 1) compared to cultures immediately extracted after inoculation.

We also examined whether *C. vaccinii* not only produces, but also excretes FR*.* For this, we investigated the FR content in both, the pellet and the supernatant of *C. vaccinii* grown in LB medium. The supernatant contained a significantly (*paired t-test*: *p* < 0.0001) higher FR concentration as compared to the pellet (Fig. 2b). It is thus concluded, that under soil-like conditions the FR concentration is between 0.00010 mg/mL (SESOM alone) and 0.00007 mg/mL (SESOM plus chitin). Furthermore, FR is excreted by *C. vaccinii*, suggesting the presence of FR in soil inhabited by *C. vaccinii*.

### Nematode G_q_ Proteins and their FR-binding Site

Nematodes are ubiquitously distributed in soil (Bardgett and van der Putten 2014; van den Hoogen et al. 2019) and many of them feed on bacteria. Therefore, it is conceivable that FR is produced as a defense *inter alia* against nematodes by soil bacteria, such as those belonging to the genus *Chromobacterium* (Taniguchi et al. 2003a; Hermes et al. 2021b). Nematodes are also dreaded plant pathogens, which may be similarly affected by FR. This is quite plausible, since G_q_ proteins are highly conserved in most eucaryotes (Mendoza et al. 2014; Lokits et al. 2018).

To date, no data are available to judge the FR sensitivity of nematode G_q_ proteins. Hence, we searched for available G_q_ protein sequences from nematodes and aligned the respective putative FR-binding sites. Such *in silico* evaluations were thought to be very insightful, since the binding site of the FR related YM to the human G_q_ protein is known in detail and in parts confirmed for FR via mutagenesis and binding studies (Malfacini et al. 2019; Voss et al. 2021).

The bacteria-feeding nematode *C. elegans* is a well-studied model organism (Brenner 1974; Harris et al. 2020; Davis et al. 2022). It expresses a Gα_q_ ortholog with 82% amino acid sequence identity to the murine Gα_q_, as deduced from cDNA sequences. *C. elegans* Gα_q_ is encoded by *egl-30*, and the respective protein plays a crucial role in nematode physiology, e.g., egg-laying, locomotion, pharyngeal activity, and axon regeneration (Brundage et al. 1996). To find further nematode Gα_q_ sequences, we conducted a search with the Basic Local Alignment Search Tool (BLAST) (Altschul et al. 1997, 2005) using the amino acid sequence of the Gα_q_ isoform a of *C. elegans* (UniProt: G5EGU1), and restricting the organism group to the taxon Nematoda. The output (100 sequences) represented mostly sequences of parasitic nematodes known to infect humans and mammals with no direct connection to soil, and was consequently filtered, selecting only nematodes belonging to the genus *Caenorhabditis* (9 sequences) or being associated with soil (8 sequences). Additionally, the genome and transcriptome of *H. schachtii* were sequenced (Siddique et al. 2022) and subsequently searched by for probable Gα_q_ orthologs. This way an mRNA consisting of 353 amino acids was identified and used for the alignment (Access to the sequences was provided to us by Dr Sebastian-Eves-van den Akker). A summary of all organisms used for the alignment can be found in supplementary Tab. S1 of Online Resource 1.

**Fig. 3:**
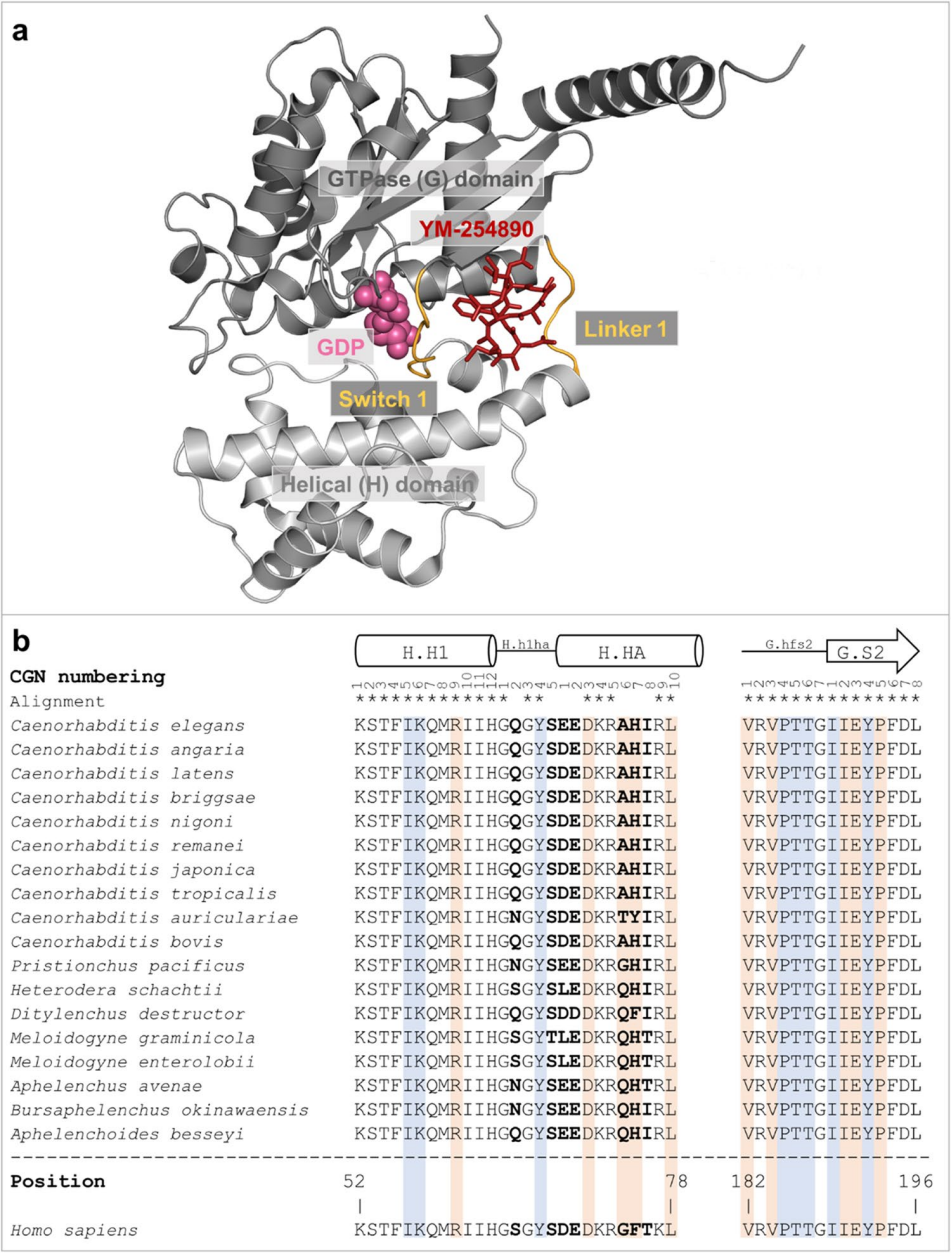
**a** Gα_i/q_βγ heterotrimer (grey) in complex with YM-254890 (red, sticks) and guanosine diphosphate (spheres, rose) (PDB Code: 3AH8). Linker 1 and switch I (linker 2) are shown in orange. **b** FR- and YM-binding sites in Gα_q_ proteins of nematodes. Sequences (Supplementary Tab. S1 of Online Resource 1) were aligned using the Clustal W algorithm and compared to the human Gα_q_ protein (UniProtKB: P50148). Identical positions within the binding sites are marked with asterisks and positions shown in bold are divergent. Positions predicted to be important for FR- and YM-binding are highlighted in blue, and positions confirmed via mutagenesis studies highlighted in orange (Nishimura et al. 2010; Malfacini et al. 2019; Voss et al. 2021). The Gα_q_ nomenclature from CGN was used and secondary structures were indicated with symbols (cylinder = α-helix; arrow = β-sheet) (Flock et al. 2015)

G_q_ proteins are highly conserved in metazoa and essential for their life (Mendoza et al. 2014; Lokits et al. 2018). Gα_q_, Gα_11_, Gα_14_, and Gα_16_ belong to the Gα_q_ family and FR potently inhibits all of them, except Gα_16_. The putative FR-binding site has been investigated using the crystal structure of the FR-related depsipeptide YM (Fig. 1) with G_q_ (Fig. 3a) (Nishimura et al. 2010) and further mutagenesis and binding studies (Malfacini et al. 2019; Voss et al. 2021). Taken together, these studies show that FR binds to human G_q_ in the interdomain cleft between the helical (H) and GTPase domain (G) by interacting with linker I and switch I (linker 2) (Voss et al. 2021). It has been revealed that hydrophobic interactions are important for FR-binding, including positions in the human Gα_q_ according to CGN nomenclature (Flock et al. 2015), V182^G.hfs2.1^ and V184^G.hfs2.3^, I190^G.S2.2^, E191^G.S2.3^, and P193^G.S2.5^, followed by, G74^H.HA.6^, F75^H.HA.7^, and L78^H.HA.10^. Furthermore, the polar aspartate D71^H.HA.3^is crucial for a salt bridge formation with R60^H.H1.9^, which stabilizes FR-binding via hydrogen bonds (Nishimura et al. 2010; Malfacini et al. 2019; Voss et al. 2021). Apart from these amino acid residues, eight others were predicted to directly or indirectly interact with YM and therefore, may also be relevant for FR-binding (Nishimura et al. 2010).

**Fig. 4:**
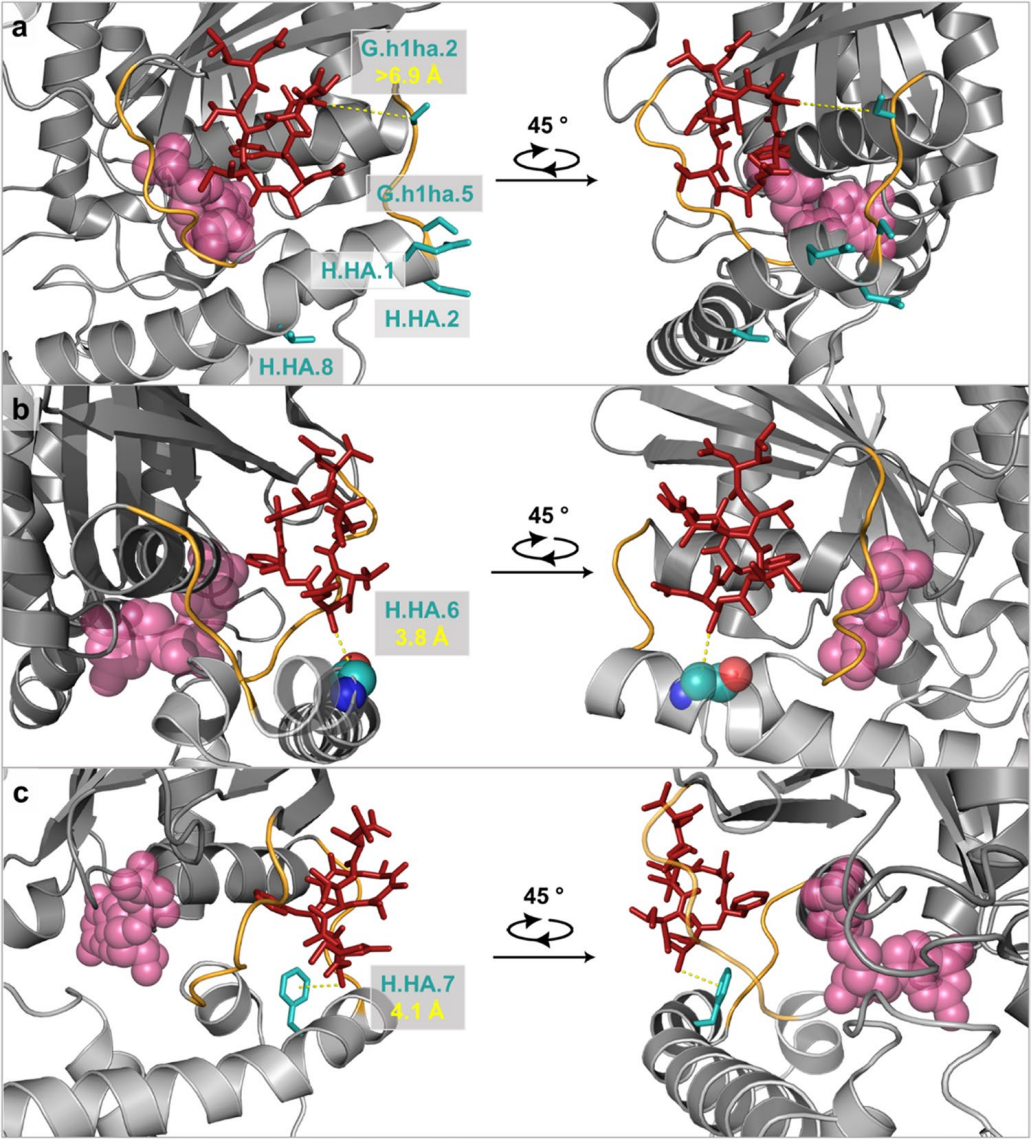
Gα_i/q_βγ heterotrimer (grey) in complex with YM-254890 (red, sticks) and guanosine diphosphate (spheres, rose) (PDB Code: 3AH8). Linker 1 and switch I (linker 2) are shown in orange. Representation of human residues at positions with differing nematode amino acids in the Gα_q_ sequence alignment S65^G.h1ha.2^, S68^G.h1ha.5^, D69^H.HA.1^, E70^H.HA.2^, and T76^H.HA.8^ in **a** as turquoise sticks; G74^H.HA.6^ in **b** as spheres; F75^H.HA.7^ in **c** as turquoise sticks. Each position was rotated once by 45° along the z-axis. Measured distances between the amino acids and YM are shown as dashed yellow lines. Visualization and measurement were done using PyMOL™ 2.5.4

In our alignment, we compared 42 amino acid residues of the putative FR-binding region of the human Gα_q_ protein with the corresponding sequences in nematode Gα_q_ proteins to identify similarities and differences (Fig. 3b). To estimate the importance of any difference in the sequences of the FR-binding sites, the inhibitor binding was visualized in the YM-G_q/i_ crystal structure (PDB code: 3AH8) using PyMOL™ 2.5.4 (Fig. 4) (Nishimura et al. 2010).

Within the 42 amino acid residues of the putative FR- and YM-binding site (Fig. 3), we identified seven positions which differ between the Gα_q_ of nematodes as compared to the FR-sensitive human Gα_q_ protein: position G.h1ha.2, G.h1ha.5, H.HA.1–2, and H.HA.6–8 (Fig. 3b). As shown in Fig. 4a the S65^G.h1ha.2^ residue is distant to the inhibitor (> 6.9 Å), and a change at this position will most likely not affect FR- and YM-binding as previous studies suggest. In a similar way the changes at positions S68^G.h1ha.5^, D69^H.HA.1^, E70^H.HA.2^, and T76^H.HA.8^ as depicted in Fig. 4a, are unlikely to influence FR- or YM-binding markedly, as the respective residues point away from the inhibitor, which is in line with studies already published (Nishimura et al. 2010).

A more profound influence on FR- and YM-binding might arise from changes in positions G74^H.HA.6^ and F75^H.HA.7^. While there is a glycine residue at H.HA.6 in the human Gα_q_ protein, most nematode Gα_q_ proteins contain an alanine or glutamine residue at this position, which represents a considerable change. Figure 4b illustrates that the distance between glycine in the heterotrimeric human G_i/q_ chimeric protein and YM is 3.8 Å and changes at this position might influence FR- and YM-binding. Former experiments substantiate this assumption, as exchanging glycine to valine at this position leads to a faster dissociation of an FR-based radioligand [^3^H]PSB-15900-FR (Voss et al. 2021).

F75^H.HA.7^ is predicted by Nishimura et al. to directly interact with the FR congener YM (Nishimura et al. 2010), which is further supported for FR itself (Voss et al. 2021). F75^H.HA.7^ is part of a hydrophobic network important to stabilize FR- and YM-binding and has approximately 4.1 Å distance to YM as predicted from the crystal structure (Fig. 4c). Our alignment revealed that 16 out of 18 sequences of nematodes contain histidine at H.HA.7, and all 18 Gα_q_ sequences of nematodes (Fig. 3b) contain amino acids with aromatic residues, e.g., tyrosine, histidine or phenylalanine. As neither of these amino acids disrupts the hydrophobic network, this change is not considered to severely impact FR-binding.

Regions G.hfs2 and G.s2 (Fig. 3) of all Gα_q_ proteins are clearly involved in YM- and FR-binding (Nishimura et al. 2010; Malfacini et al. 2019; Voss et al. 2021) and are identical between the Gα_q_ proteins of all selected nematodes and the human ortholog.

Taken together, the Gα_q_ proteins of nematodes are likely to be inhibited by FR and YM. There are two positions in Gα_q_ proteins of nematodes which differ from human Gα_q_ and possibly influence the binding of FR. To substantiate this further, we used *in vitro* investigations with Gα_q_ of the nematode model organism *C. elegans* and the plant-parasitic *H. schachtii*.

Comparing both organisms used for the *in vitro* Gα_q_ inhibition assay with FR, i.e., *H. schachtii* and *C. elegans*, a pairwise alignment of both full length Gα_q_ amino acid sequences revealed 90% identity. Looking at the investigated FR-binding region of these two nematodes (Fig. 3) only three positions differ, G.h1ha.2, H.HA.1, and H.HA.6. As pointed out above the first two positions, G.h1ha.2 and H.HA.1, are supposedly not required for the interaction with the inhibitor (Fig. 4a), and only the difference at position H.HA.6 is seemingly of interest. Plant parasitic nematodes like *H. schachtii* have a more spacious residue with glutamine at position H.HA.6 compared to the alanine present in nematodes belonging to the *Caenorhabditis* group. As it is difficult to predict the influence of these changes on the binding and activity of FR, we investigated the inhibition of FR on nematode Gα_q_ *in vitro*.

### FR Inhibits Heterologously Expressed Gα_q_ Proteins of *C. elegans* and *H. schachtiiin vitro*

To verify our bioinformatic predictions, we chose *H. schachtii* as representative for the plant pathogenic nematodes and *C. elegans* as representative for the bacteria-feeding nematodes and well-characterized model organism to investigate the sensitivity of these two nematode Gα_q_ isoforms to FR *in vitro*.

The Gα_q_ protein of *H. schachtii* was transiently introduced into HEK293 cells genetically edited with CRISPR/Cas9 technology to lack endogenous G_q_ and G_11_ proteins (HEK G_q/11_-KO cells) to avoid signal confounding. As functional G_q_ readout, we measured the accumulation of inositol monophosphate (IP_1_) after stimulation of the endogenously expressed muscarinic acetylcholine receptor type 3 (M3) with carbachol. However, even at concentrations as high as 100 µM carbachol, expression of *H. schachtii* Gα_q_ did not increase the level of IP_1_ over vector control, whereas expressing murine G_q_ resulted in increased IP_1_ accumulation under the same conditions.

**Fig. 5:**
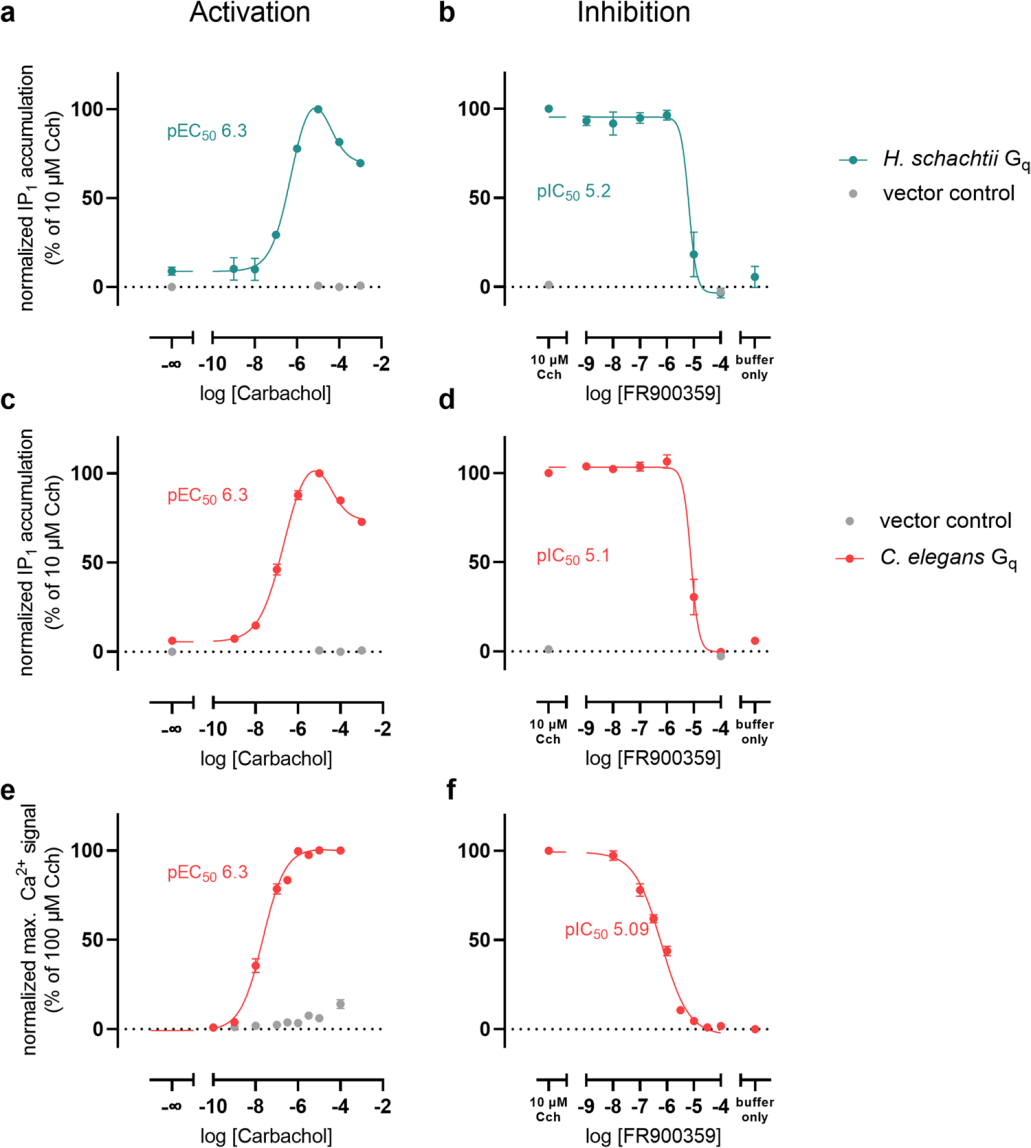
Functional expression of nematode Gα_q_ proteins and inhibition by FR. **a**, **c** Concentration-dependent IP_1_ accumulation after stimulation of HEK293 Gα_q_/Gα_11_-null cells transfected to express **a** *H. schachtii* and **c** *C. elegans* Gα_q_ isoforms with carbachol. **b**, **d** Concentration-inhibition curves of FR on *H. schachtii* **b** and *C. elegans* **d** Gα_q_ proteins normalized to the IP_1_ accumulation evoked by 10 µM carbachol. **e** Concentration-dependent calcium signal after stimulation of HEK293 Gα_q_/Gα_11_-null cells transfected to express *C. elegans* Gα_q_ isoforms with carbachol. **f** Concentration-inhibition curves of FR on *C. elegans* Gα_q_ proteins normalized to the calcium signal evoked by 100 µM carbachol. Mean ± SEM, at least 3 biological replicates performed in triplicate

The lack of signal might be due to various reasons, ranging from improper protein folding or location to the missing of further proteins necessary for *H. schachtii* G_q_ signaling. One of these proteins might be resistant to inhibitors of cholinesterase 8A (RIC-8A), which was first identified as a crucial part of G_q_ signaling in the nematode *C. elegans* (Miller et al. 2000). Later, RIC-8 was shown to facilitate G protein folding and thereby expression (Chan et al. 2013) and to act as guanine-nucleotide exchange factor (GEF) (Tall et al. 2003). Moreover, it has previously been used to amplify signaling of other insect G proteins (Himmelreich et al. 2017). Based on this, we decided to co-express RIC-8A with *H. schachtii* Gα_q_ to increase expression and enable signaling. However, RIC-8A co-expression alone did not result in a measurable response of the Gα_q_ protein to carbachol. In a further attempt, we over-expressed the M3 receptor for G protein activation, which again, on its own, did not lead to an increase in IP_1_ accumulation over vector control (Supplementary Fig. S3). Only the combination of both, RIC-8A and M3 expression, led to a concentration-dependent IP_1_ accumulation upon carbachol addition as shown in Fig. 5a.

To investigate the FR sensitivity of *H. schachtii* Gα_q_, we pre-incubated cells with varying concentrations of the inhibitor and stimulated with 10 µM carbachol as this concentration elicits the highest response. In line with our predictions from the alignment, FR was able to completely blunt *H. schachtii* G_q_ signaling with low micromolar potency (Fig. 5b).

To investigate the Gα_q_ protein of *C. elegans*, we repeated the activation and inhibition experiments with HEK G_q/11_-KO cells transiently expressing *C. elegans* Gα_q_ cDNA. As is evident from Fig. 5c, *egl-30* can be functionally expressed in HEK G_q/11_-KO cells when following the established protocol of over-expressing M3 and RIC-8A. Moreover, the activation behavior of *C. elegans* Gα_q_ closely resembles that of *H. schachtii* Gα_q_, considering that the shapes of the dose response curves are similar and the pEC_50_ values are comparable. Regarding inhibition, *C. elegans* Gα_q_ is clearly FR-sensitive with full inhibition at 100 µM inhibitor and an IC_50_ value in the low micromolar range (Fig. 5d).

*C. elegans* is a well-known nematode model organism with a plethora of established experimental methods available, which allows studying FR effects more easily in this organism. Therefore, we were inclined to corroborate our findings with an additional read-out for the assessment of *C. elegans* Gα_q_ activity. We chose to repeat the same set of activation and inhibition experiments now measuring the increase of intracellular calcium concentrations as consequence of G_q_ activation. Again, functional expression was achieved (Fig. 5e). Remarkably, when inhibiting with FR (Fig. 5f), the resulting dose–response curve was shifted to the left to a high nanomolar potency, showing a more moderate steepness of the curve compared with the IP_1_ accumulation assay. These results confirm the ability of FR to inhibit *C. elegans* Gα_q_.

Taken together, we were able to express functionally active Gα_q_ proteins of *H. schachtii* and *C. elegans* in cell cultures and confirmed their inhibition by FR. Interestingly, both *H. schachtii* and *C. elegans* Gα_q_, were inhibited with a similar potency in the IP_1_ assay, implicating that the difference in the FR-binding site between both proteins, alanine as opposed to glutamine at position H.HA.6 is not interfering with FR activity, conceivable because a glutamine at this position might face away from the inhibitor. These promising *in vitro* results made *in vivo* effects very likely, which were thus the focus of our next experiments.

### FR Impairs Movement, Spatial Distribution, and Egg-laying of *C. elegansin vivo*

To investigate the effect of FR on Gα_q_ of *C. elegans*, we used *C. elegans* wildtype N2 and a set of three strains with different *egl-30* mutations to illustrate G_q_ loss-of-function phenotypes. The strong *egl-30*(*ad805*) mutant exhibits a severe egg-laying phenotype as it is nearly paralyzed and bloated with eggs, subsequently called “strong mutant”. Two hypomorphic mutants, *egl-30*(*n686*) and *egl-30*(*ad806*), are less bloated with eggs and sluggish to very sluggish, but never paralyzed, and termed “weak mutants” in this study (Brundage et al. 1996). Additionally, the G_q_ signaling suppressor mutants *dgk-1*(*sy428*) and *eat-16*(*sa609*) were utilized as opposing phenotypes. The suppressor mutant *dgk-1*(*sy428*) encodes a loss-of-function diacylglycerol kinase (DGK-1) (Jose and Koelle 2005). DGK-1 is known to act as a negative regulator of G_q_ signaling, as it phosphorylates the second messenger diacylglycerol (DAG) (Miller et al. 1999). The suppressor mutant *eat-16*(*sa609*) is a missense loss-of-function mutant of *eat-16*. The latter encodes a negative regulator of G protein signaling, which belongs to the family of GTPase activating proteins (GAPs). It leads to hydrolysis of GTP and this way turns G proteins into their inactive state (Hajdu-Cronin et al. 1999). Both suppressor mutants display hyperactivity with fast movements and rapid egg-laying (Hajdu-Cronin et al. 1999).

**Fig. 6:**
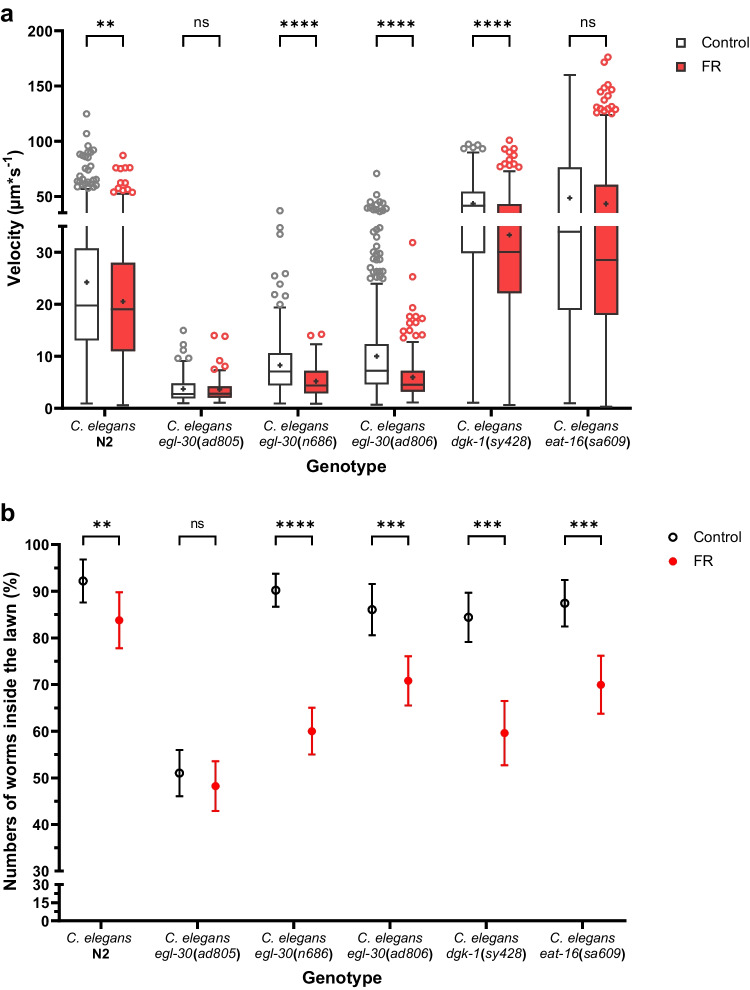
Effect of FR on velocity **a** and spatial distribution **b** of six *C. elegans* genotypes (N2, *egl-30*(*ad805*), *egl-30*(*n686*), *egl-30*(*ad806*), *dgk-1*(*sy428*), *eat-16*(*sa609*)). All nematodes were fed with *E. coli* OP50, placed as lawn in the middle of the NGM plate and mixed with 1% DMSO (Control). In the FR group 2.5 mM FR were added to the food. The movement of adult nematodes in and around the lawn was recorded and analyzed. Velocities are displayed as box plot with Tukey whiskers (Quartile ± 1.5*inter-quartile distance (IQR)), and the mean is displayed as + in **a**. Circles above and below represent outliers. All experiments except spatial distribution experiments for *egl-30*(*ad805*) (two repeats) were done in three repeats. Velocities of *C. elegans* N2, *dgk-1*(*sy428*), and *eat-16*(*sa609*) were compared (FR versus Control) using the *Mann–Whitney test* and velocity of *egl-30* mutants were compared using the *unpaired t-test*. The spatial distribution of nematodes in the lawn was compared using the *modified two sample binomial test* (Wong et al. 2014) and the empirical standard deviation was presented as error bars. *P* > 0.05 = ns, *P* < 0.05 = *, *P* < 0.01 = **, *P* < 0.001 = ***, *P* < 0.0001 = ****

Gα_q_ is important for the locomotion of *C. elegans* (Brundage et al. 1996). Therefore, tracking experiments on agar plates were performed with nematodes raised as explained in the experimental section. Three repeats were performed for each of the six genotypes (*C. elegans* N2, *egl-30*(*ad805*), *egl-30*(*n686*), *egl-30*(*ad806*), *dgk-1*(*sy428*), *eat-16*(*sa609*)) and summarized in Fig. 6. Single tracking results can be found in supplementary Fig. S4-S9 of Online Resource 1. The experimental set up included a spot of *E. coli* OP50 bacteria in the middle of the agar plate (bacterial lawn on which the nematodes feed) and allowed to analyze the velocity of nematodes and the spatial distribution of nematodes outside or in the bacterial lawn.

The velocity (Fig. 6a) of the controls (i.e., without FR treatment) of each genotype showed that both suppressor mutants, i.e., *dgk-1*(*sy428*) and *eat-16*(*sa609*), were the fastest moving genotypes, followed by the wildtype N2. Compared to that, all *egl-30* mutants, i.e., *egl-30*(*n686*), *egl-30*(*ad806*), and *egl-30*(*ad805*), were slower in movement. These results were consistent with the results from previous reports (Brundage et al. 1996; Hajdu-Cronin et al. 1999).

For the wildtype N2, FR reduced the average velocity significantly (*Multiple Mann–Whitney tests*: *p* = 0.0076) (Fig. 6a). The spatial distribution of *C. elegans* N2 was also affected by FR (Fig. 6b) as significantly less nematodes were inside the lawn, i.e., 92.2% without FR compared to 83.8% with FR (*Modified two sample binomial test* (*MBT*): *p* = 0.0021). All these results agree with the expected outcome of G_q_ inhibition.

FR did not affect the mean velocity (*Unpaired t-test: p* = 0.9876) of the strong mutant *egl-30*(*ad805*) or its spatial distribution (*MBT*: *p* = 0.6818) (Fig. 6). In this case, the data from one repeat were excluded from the analysis of the spatial distribution as the bacterial lawn of this repeat was enlarged unevenly (Supplementary Fig. S6b of Online Resource 1). Due to the already strongly impaired G_q_ signaling in this mutant strain, further G_q_ inhibition was not envisioned to have a major impact. These results are thus as expected.

For both weak *egl-30* mutants, *egl-30*(*n686*) and *egl-30*(*ad806*), FR reduced the mean velocity significantly (*Unpaired t-test*: both *p* < 0.0001) (Fig. 6a). In comparison, *egl-30*(*ad806*) had a significantly higher velocity than *egl-30*(*n686*) in the control experiment (*unpaired t-test*: 0.0168), which was in line with previous reports (Brundage et al. 1996). The spatial distribution of *egl-30*(*n686*) (*MBT*: *p* < 0.0001) and *egl-30*(*ad806*) (*MBT*: *p* = 0.0003) was affected significantly by FR (Fig. 6b). The results for both, *egl-30*(*n686*) and *egl-30*(*ad806*), were as expected, as the inhibition of G_q_ by FR increased their impairment leading to similar phenotypes as observed for *egl-30*(*ad805*).

The suppressor mutant *dgk-1*(*sy428*) depicted a significantly reduced velocity in presence of FR (*Mann–Whitney tests*: *p* < 0.0001) (Fig. 6a). This was expected as FR reduced G_q_ hyperactivity caused by the mutation *of dgk-1*. Compared to N2 in presence of FR, *dgk-1*(*sy428*) had a significantly higher velocity in presence of FR (*Mann–Whitney test*: *p* < 0.0001) meaning that *dgk-1*(*sy428*) was able to rescue the velocity decrease by FR. The spatial distribution was significantly affected by FR, as less worms stayed inside the FR containing bacterial lawn (*MBT*: *p* < 0.0001) (Fig. 6b).

The suppressor mutant *eat-16*(*sa609*) displayed a slightly different picture, as there was a reduction of velocity, which was however not significant (*Multiple Mann–Whitney tests*: *p* = 0.0534). However, *eat-16*(*sa609*) grown in presence of FR was able to rescue the velocity decrease of N2 grown in presence of FR (*Mann–Whitney test*: *p* < 0.0001) as expected. Concerning spatial distribution, the number of nematodes inside the lawn was significantly decreased in presence of FR (*MBT*: *p* < 0.0001) (Fig. 6). Compared to *dgk-1*(*sy428*) a greater span of velocities was observed for *eat-16*(*sa609*), which might be the reason for the lack of significance.

All nematodes, except the strong *egl-30*(*ad805*) mutant, seemed to avoid the presence of FR (Fig. 6b), as the number of nematodes was always significantly lower in the bacterial lawn with FR compared to the control. If *C. elegans* detects FR via its sensory system and initiates lawn avoidance as described for serrawettin W2, a cyclic depsipeptide and biosurfactant produced by *Serratia marcescens* Db10 (Pradel et al. 2007), it is likely that FR-Core would be detected similarly. To study whether FR could affect the food quality for *C. elegans*, we did experiments with FR-Core that did not result in a changed spatial distribution of *C. elegans* N2 (Supplementary Tab. S2 of Online Resource 1), which suggests that avoidance is a result of FR’s inhibitory effect. G_q_ signaling plays an antagonistic role in olfactory adaption to AWC-sensed odorants (Matsuki et al. 2006). Therefore, the G_q_ inhibition by FR might increase avoidance as nematodes would adapt faster. Nevertheless, as avoidance has been observed for all genotypes, except the strong mutant *egl-30*(*ad805*), the effect of FR is probably not solely connected to G_q_ inhibition. Further studies investigating a learned avoidance of FR by *C. elegans* might reveal new insights into the interaction of FR targets and FR producers.

As Gα_q_ malfunction is associated with defective egg-laying (i.e., *egl*) by *C. elegans* (Brundage et al. 1996), we examined this in the presence of FR. In the respective set of experiments the strong *C. elegans egl-30*(*ad805*) mutant was not included, as time extended tracking experiments already had shown, as expected, that this mutant was not at all able to lay eggs (Supplementary Fig. S10 of Online Resource 1). To examine the egg-laying rate, all five genotypes (*C. elegans* N2, *C. elegans egl-30*(*n686*), *C. elegans egl-30*(*ad806*), *C. elegans dgk-1*(*sy428*), *C. elegans eat-16*(*sa609*)) were raised as described in the experimental section. Once they started to lay eggs, 12 nematodes per genotype and for each condition (i.e., with and without FR) were picked and placed on a new plate with the same condition (i.e., with or without FR). After two hours, nematodes were removed, and the eggs laid were counted.

**Fig. 7:**
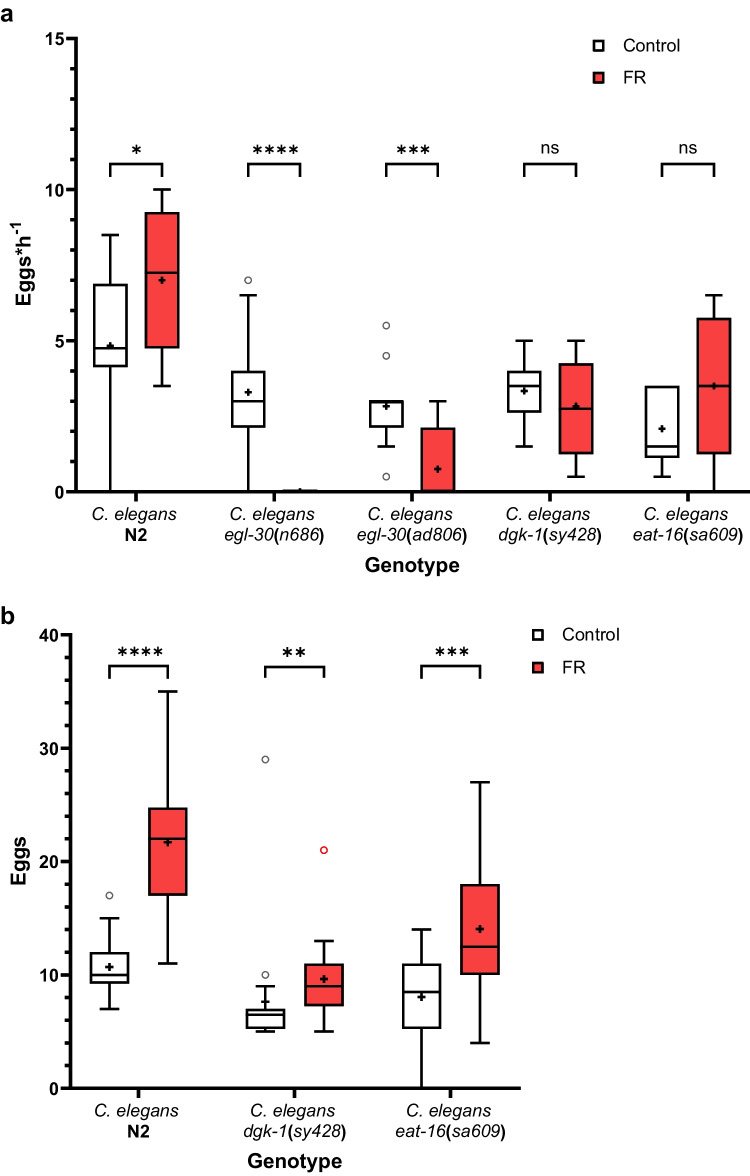
FR inhibits egg-laying of *C. elegans*. **a** Egg-laying rate for different *C. elegans* strains (Wildtype/N2, *egl-30*(*n686*), *egl-30*(*ad806*), *dgk-1*(*sy428*), and *eat-16*(*sa609*)) in presence (FR) or absence of FR (control). Both groups were grown until egg-laying adult stage on NGM plates covered with *E. coli* OP50 as food source mixed with 1% DMSO, and FR for the FR group. 12 nematodes per group were analyzed individually for their egg-laying (Worms on FR were analyzed in presence of FR and similar for control). **b** Retained egg assay was performed with *C. elegans* N2, *dgk-1*(*sy428*), and *eat-16*(*sa609*)). Both groups were grown until egg-laying adult stage on NGM plates covered with *E. coli* OP50 as food source and 1% DMSO. For the FR group 2.5 mM FR were added to the food source. 20 nematodes per group were bleached and eggs were counted. Both **a** and **b** are presented as box and Tukey whiskers (Quartile ± 1.5*inter-quartile distance (IQR)) plot. The significance was evaluated using *uncorrected multiple t-tests* for **a** and *C. elegans* N2 and *eat-16*(*sa609*) in **b**, while *dgk-1*(*sy428*) in **b** was evaluated using *Mann–Whitney test. P* > 0.05 = ns, *P* < 0.05 = *, *P* < 0.01 = **, *P* < 0.001 = ***, *P* < 0.0001 = ****

According to literature, an adult hermaphrodite *C. elegans* lays between four to ten eggs per hour (Lints and Hall 2004). This was confirmed during this experiment for the wildtype N2 with a mean of five eggs per hour (Fig. 7a). Unexpectedly, in the presence of FR the egg-laying rate of N2 slightly, but significantly increased (*Unpaired t-test*: *p *= 0.0350).

Mutation of *egl-30* result in egg-laying malfunction (Brundage et al. 1996), which was confirmed in our experiments (Fig. 7), as the weak *egl-30* mutants *egl-30*(*n686*) and *egl-30*(*ad806*) laid fewer eggs compared to wildtype. For both weak *egl-30* mutants, the egg-laying rate was significantly reduced (*Unpaired t-tests*: *p *< 0.0001, *p *= 0.0005) by FR treatment, and in the case of *egl-30*(*n686*) no eggs were laid, when FR was present. These results agree with the expected outcome, considering the influence of G_q_ on egg-laying.

The suppressor mutants *dgk-1*(*sy428*) and *eat-16*(*sa609*) showed reduced egg-laying compared to N2, which might be caused by the lack of egg production (Hajdu-Cronin et al. 1999). The suppressor mutants, *dgk-1*(*sy428*) and *eat-16*(*sa609*) were not significantly affected by FR treatment (*Unpaired t-tests*: *p *= 0.3542, *p *= 0.0618).

Our results show that FR treatment caused a much more severe phenotype for the two weak *egl-30* mutants *egl-30*(*n686*) and *egl-30*(*ad806*). In addition, the two suppressor mutants *dgk-1*(*sy428*) and *eat-16*(*sa609*) were not affected by FR treatment, which was consistent with their known functions as suppressors of egl-30 mutants. However, it should be noted that FR treatment on N2 showed an unexpected result of increased egg-laying, possibly because of more eggs retained, or due to multifactorial influences.

A more common approach to investigate egg-laying defects is to count the numbers of eggs in the uterus. To disentangle the effects on egg-laying versus egg production, we performed the retained eggs assay and counted the number of eggs in the uterus of a worm, reasoning that the more eggs are still in the uterus, the lower must be the egg-laying rate. The weak *egl-30* mutants, *egl-30*(*n686*) and *egl-30*(*ad806*), were not evaluated again, due to the clear and unequivocal result in the egg-laying assay (Fig. 7a). To examine the number of retained eggs, nematodes were raised as detailed in the experimental section. After they reached the egg-laying adult stage, 20 worms per condition (i.e., with and without FR) and genotype (*C. elegans* N2, *C. elegans dgk-1*(*sy428*), *C. elegans eat-16*(*sa609*)) were picked and dissolved in bleach solution. Afterwards the remaining eggs were counted.

As depicted in Fig. 7b, wildtype N2 nematodes grown in presence of FR had significantly more eggs (*Unpaired t-test*: *p *< 0.0001) in their uterus. Untreated *eat-16*(*sa609*) and *dgk-1*(*sy428*) had a mean of 8±1 eggs in their uterus. Prior reports had found fewer retained eggs, but used a different timing (Hajdu-Cronin et al. 1999). FR treatment increased the number of eggs in the mutants *dgk-1*(*sy428*) (*Mann-Whitney test*: *p *= 0.0012) and *eat-16*(*sa609*) (*Unpaired t-test*: *p *= 0.0005) as well. These results were as expected, as G_q_ inhibition by FR led to higher numbers of eggs in the uterus due to egg-laying inhibition. Additionally, both suppressor mutants were able to rescue N2 grown in presence of FR as the number of eggs in the uterus was significantly decreased by *dgk-1*(*sy428*) (Mann-Whitney test: *p *< 0.0001) and *eat-16*(*sa609*) (unpaired t-test: *p *= 0.0001).

Taken together, FR reduced velocity and inhibited egg-laying of *C. elegans.* Our results clearly show that FR targets the Gα_q_ ortholog of *C. elegans* and leads to phenotypes as observed for *egl-30* deficient mutants.

### FR Reduces *H. schachtii* J2 Activity and Inhibits Hatching from Cysts

To assess the effect of FR on plant pathogenic nematodes, the cyst nematode *H. schachtii* was assayed *in vivo*. Parasitic cyst nematodes, e.g., *Globodera* and *Heterodera* spp., are difficult to erase from soil, as unhatched juvenile stage 2 worms (J2) can survive in the cyst for a long time. After hatching, J2 worms search the root of a plant which they penetrate, migrate into, and form a syncytium by modifying plant cells. Feeding on the syncytium, nematodes mature into the J4 stage for males or into adult stage for females. Fertilized females develop into the cysts. During the life cycle of *H. schachtii* only two stages are detached from the host plant, i.e. the cyst and the J2 stage (Sijmons 1993; Lilley et al. 2005; Bohlmann and Sobczak 2014; Ngala et al. 2020).

**Fig. 8:**
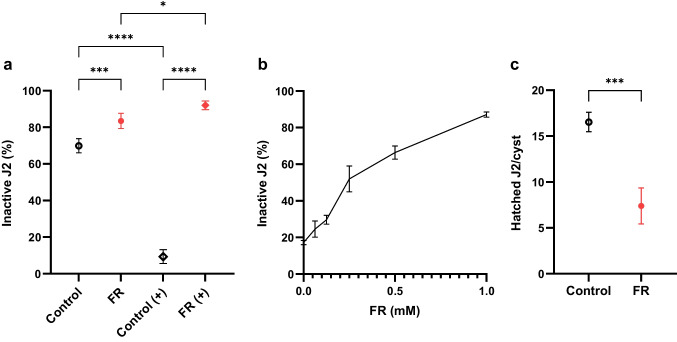
Effect of FR on movement **a**, **b** and hatching **c** of *H. schachtii* J2. **a** After incubation of *H. schachtii* J2 for 4 days in the presence of FR (1 mM) mixed with 1% DMSO (FR) or 1% DMSO (control) either with ( +) or without octopamine the number of inactive (not bend or moving) and active worms was counted. The relative amount is depicted on the y-axis. The significance was evaluated using ordinary *One-way ANOVA with Tukey's multiple comparisons test*. **b** After incubation of *H. schachtii* J2 for 4 days in presence of six different FR concentrations (0, 0.0625, 0.125, 0.25, 0.5, 1 mM) mixed with 1% DMSO the number of inactive (not bend or moving) and active worms was counted. The relative amount is depicted on the y-axis. **c** Comparison of hatched *H. schachtii* juveniles per cyst with 1 mM FR disolved in 1% DMSO (FR) or 1% DMSO (control) after incubation of 7 days. The significance was evaluated using *unpaired t-test*. All experiments, *n* = 4. *P* > 0.05 = ns, *P* < 0.05 = *, *P* < 0.01 = **, *P* < 0.001 = ***, *P* < 0.0001 = ****

Both J2s and cysts were exposed to FR and moving *versus* nonmoving nematodes and hatched nematodes were counted respectively. The experiment with J2 stage nematodes was conducted with and without octopamine, which is used to stimulate feeding leading to an oral uptake of xenobiotic compounds from surrounding media (Urwin et al. 2002). Compared to the control, FR (without octopamine) significantly increased the number of inactive nematodes from 70 to 83% (*One-way ANOVA*: *p* = 0.0009). This effect was, however, even more pronounced in the presence of octopamine with only 9% inactive nematodes in the control and 92%, respectively in the presence of FR (*One-way ANOVA*: *p* < 0.0001). Octopamine alone significantly lowered the number of inactive nematodes in the control experiment from 70 to 9% (*One-way ANOVA*: *p* < 0.0001) and led to a significant increase of inactive worms in presence of FR from 83 to 92% (*One-way ANOVA: p* = 0.0262) (Fig. 8a). These observations were expected as octopamine increased the uptake of xenobiotic compounds like FR (Urwin et al. 2002) and significantly lowered the number of inactive nematodes in the control experiment due to its influence on movement (Masler 2007) and quiescence (Griffin and Fitters 2004; Schroeder and MacGuidwin 2010). These results show that the inhibition of G_q_ by FR leads to a decreased activity of *H. schachtii* J2, the physiology behind this is however unknown.

Next, we investigated the concentration-dependency of this effect. As Fig. 8b depicts the number of inactive nematodes at the J2 stage is increasing in a FR concentration-dependent manner.

We then exposed cysts of *H. schachtii* to FR and counted the number of hatched juveniles in comparison to not treated cysts (Fig. 8c). FR inhibited hatching of J2, as the number of hatched J2 per cyst decreased in the presence of FR from 17 ± 1 to 7 ± 1 hatched J2 per cyst (*Unpaired t-test*: *p* = 0.0002).

Taken together, inhibition of G_q_ by FR leads to an inhibition of hatching from the cysts of *H. schachtii* J2. Hatching from cysts requires movement of the juvenile worms (Wallace 1968) and since FR inhibits such activity, J2 may be unable to hatch. Other explanations are also possible, e.g., an influence of FR on the development of *H. schachtii* stages or inhibition of signaling cascades triggering hatching.

## Discussion

Microorganisms living in soil form an extremely complex microbiome, which plays a significant role in our ecosystem affecting the health of plants, animals and humans, one of the phenomena called “one health” (Banerjee and van der Heijden 2023). The challenge to maintain a healthy and functioning soil microbiome depends on our knowledge of this complex system and its interactions (Jansson and Hofmockel 2020; Banerjee and van der Heijden 2023). Disease suppressive soils, in which fungi and bacteria cooperate to suppress plant parasitic nematodes demonstrate the beneficial interactions of the microbiome (Topalović et al. 2020). Research focusing on the soil microbiome is faced with an enormous complexity and huge gaps of knowledge (Fierer 2017; Mishra et al. 2022). The herein described investigation of the role of a bioactive natural product like FR and its microbial producer *C. vaccinii* contributes to decipher the complex puzzle of the soil microbiome.

*C. vaccinii* is not only producing FR, but also other secondary metabolites like the nonribosomal lipopeptides valhidepsins (Pistorius et al. 2023) and the purple pigment violacein (Soby et al. 2013). Valhidepsin-1 functions as surfactant (Pistorius et al. 2023) and may be required for the mobility of *C. vaccinii* on solid surfaces (Gutiérrez-Chávez et al. 2021). As the BGCs of valhidepsins and FR are co-localized, a synergistic effect in the ecological context is reasonable (Pistorius et al. 2023). Violacein itself is known as a quorum sensing indicator as the adaptive response triggered by the required quorum of bacteria leads to the production of this purple pigment (Durán et al. 2016; Park et al. 2021). Multiple biological effects of violacein have been investigated (Durán et al. 2021) including nematocidal activity (Ballestriero et al. 2014, 2016). Synergistic experiments investigating the effect of these compounds together with FR may even more profoundly decipher the role of *C. vaccinii* in soil.

We herein present data showing that *C. vaccinii* is producing FR in soil-derived liquid media, i.e., SESOM from garden soil (Fig. 2a). However, the concentrations found in SESOM were low (around 0.0001 mg/mL) probably due to an insufficient mimicking of natural conditions. *C. vaccinii* was originally found near the root of cranberries (*Vaccinium macrocarpon* Ait) in bog soil (Soby et al. 2013), which consists of alternating layers of sand and organic matter supposedly with other nutrients than garden soil (Putnam et al. 2003). *C. vaccinii* might also be part of the rhizosphere of *V. macrocarpon* and therefore supplied with root exudates, which are lacking in SESOM. It also must be considered that multiple organisms are interacting in soil, inducing or inhibiting the production of metabolites. These interactions are difficult to simulate, but likely to influence FR production. The potential of *C. vaccinii* to produce higher amounts of FR is obvious from cultivations in LB medium, reaching levels of 2.5 mg/L (Hermes et al. 2021b). To answer the question on how much FR is really present in certain environments, an in situ approach would be of interest, in which a suitable adsorber resin is applied to extract FR from the respective surrounding (Kudela 2011, 2017; Tuttle et al. 2019). A metagenomic analysis of environmental soil DNA for the *frs* BGC (Santana-Pereira et al. 2020) would yield novel data to judge the overall presence of potential FR producers. Still, though limited, our approach clearly indicates the ability of *C. vaccinii* to produce FR in soil.

FR is an inhibitor of G_q_ proteins involved in signal transduction of GPCRs and shown to have the potential to protect plants from insects (Crüsemann et al. 2018) and mammals (Miyamae et al. 1989; Matthey et al. 2017) *in vitro* and *in vivo*. Our *in silico* analysis of the putative FR-binding sites of various nematodic G_q_ proteins strongly indicated the possibility of FR-binding to an inhibition of these proteins. (Figs. 3 and 4). *In vitro* expression of *egl-30* (i.e. G_q_) of *C. elegans* and the here newly identified Gα_q_ ortholog of *H. schachtii* in HEK G_q/11_-KO cells, together with overexpression of RIC-8A and M3, enabled functional expression and characterization of the proteins (Fig. 5). *In vitro*, FR inhibited both, *C. elegans* and *H. schachtii* Gα_q_ signaling, as IP_1_ accumulation and calcium increase were blocked.

*In vivo*, FR affects the nematode *C. elegans* by significantly decreasing their velocity (Fig. 6b) and inhibiting egg-laying (Fig. 7). Considering that *C. elegans* is feeding on bacteria, these effects suggest that *C. vaccinii* produces FR to reduce the activity and abundance of predators. *H. schachtii* is a pathogen feared in agricultural cultivation of crops belonging to the family Brassicaceae or Amaranthaceae, especially sugar beets (*Beta vulgaris*) (Daub 2021), as the cysts persist in soil and are difficult to erase (Ngala et al. 2020). *In vivo* experiments (Fig. 8) demonstrate an inhibitory effect of FR on the activity of J2 *H. schachtii* and revealed FR to suppress hatching of J2.

It must be noted, however that the low concentrations of FR observed in SESOM could hardly lead to similar *in vivo* effects on *C. elegans* and *H. schachtii*. Chronic exposure to FR in soil and exponential effects (Chianese et al. 2018; Almasri et al. 2022) as conceivable by reduced egg-laying or hatching, could work together in a synergistic fashion. Also, a higher production of FR by *C. vaccinii* in soil compared to SESOM is reasonable. The presence of other potent metabolites from *C. vaccinii*, e.g., valhidepsins, for which a surfactant effect has been shown (Pistorius et al. 2023), and violacein (Soby et al. 2013), which is reported to have also nematocidal and antibacterial activity (Ballestriero et al. 2014) also has to be taken into account.

In this study, we provide one conclusive example for how, in the ecological context, the presence of bacteria like *C. vaccinii* and their excreted metabolome in the soil might contribute to an ecological equilibrium, being a prerequisite for the fruitful growth and cultivation of plants.

### Supplementary Information

Below is the link to the electronic supplementary material.

Supplementary file1 (DOCX 9582 kb)

Supplementary file2 (XLSX 153 kb)

## Data Availability

The data used to support the findings of this study are included in this article. The authors confirm that all other analyzed data and/or raw data generated during this study are available in supplementary information.
