# Supplementary material for: The Bacterial Gq Signal Transduction Inhibitor FR900359 Impairs Soil-Associated Nematodes
Source: J Chem Ecol. 2023 Jul 15;49(9-10):549–69. doi: 10.1007/s10886-023-01442-1 (PMC10725363; doi:10.1007/s10886-023-01442-1)
Supplement: Supplementary file 1 — Supplementary file1 (DOCX 9582 kb) [file 10886_2023_1442_MOESM1_ESM.docx]

Supporting Information

THE BACTERIAL G_q_ SIGNAL TRANSDUCTION INHIBITOR FR900359 IMPAIRS SOIL-ASSOCIATED NEMATODES

Wiebke Hanke^1,#^, Judith Alenfelder^2,#^, Jun Liu^5^, Philipp Gutbrod^3,4^, Stefan Kehraus^1^, Max Crüsemann^1^, Peter Dörmann^3^, Evi Kostenis^2^, Monika Scholz^5,#^, and Gabriele M. König^1,#,*^

*^1^Institute of Pharmaceutical Biology, University of Bonn, Nussallee 6, D-53115 Bonn, Germany.*

*^2^Molecular, Cellular and Pharmacobiology Section, Institute for Pharmaceutical Biology, University of Bonn, Nussallee 6, D-53115 Bonn, Germany.*

*^3^Institute of Molecular Physiology and Biotechnology of Plants (IMBIO), University of Bonn, Karlrobert-Kreiten-Straße 13, D-53115 Bonn, Germany.*

*^4^Current address: Bonn International Graduate School – Land and Food, University of Bonn, Katzenburgweg 9, D-53115 Bonn, Germany.*

*^5^Neural information flow, Max Planck Institute for Neurobiology of Behavior – CAESAR, Ludwig-Erhard-Allee 2, D-53175 Bonn, Germany.*

^#^These authors contributed equally to this work

*Corresponding Author: Tel: (0049)-228-733194. E-Mail: g.koenig@uni-bonn.de

[**Fig. S1** Calibration curves with the area under the curve (AUC) for FR (*m/z* 1002.5) determined by LC/MS 2](#_Toc135380095)

[**Fig. S2** FR concentration in *n*-butanol extracts of *C. vaccinii* culture in SESOM with chitin 2](#_Toc135380096)

[**Fig. S3** IP_1_ accumulation after stimulation without and with 1 mM and 10 µM carbachol (CcH) of HEK293 Gα_q_/Gα_11_-null cells transfected to express only *H. schachtii* Gα_q_ isoform **a**; *H. schachtii* Gα_q_ isoform with M3 overexpression **b**; with RIC-8A expression **c**; or with both M3 overexpression and RIC-8A expression **d** 4](#_Toc135380097)

[**Fig. S4** Tracking patterns of *C. elegans* N2 with and without FR 5](#_Toc135380098)

[**Fig. S5** Tracking patterns of *C. elegans* *egl-30*(*ad805*) with and without FR 6](#_Toc135380099)

[**Fig. S6** Tracking patterns of *C. elegans* *egl-30*(*n686*) with and without FR 7](#_Toc135380100)

[**Fig. S7** Tracking patterns of *C. elegans* *egl-30*(*ad806*) with and without FR 8](#_Toc135380101)

[**Fig. S8** Tracking patterns of *C. elegans dgk-1*(*sy428*) with and without FR 9](#_Toc135380102)

[**Fig. S9** Tracking patterns of *C. elegans eat-16*(*sa609*) with and without FR 10](#_Toc135380103)

[**Fig. S10** Egg-laying adults of *C. elegans egl-30*(*ad805*) after 79 hours on plate 11](#_Toc135380104)

[**Fig. S11** ^1^H NMR spectrum of FR in CDCl_3_ (600 MHz) 11](#_Toc135380105)

[**Fig. S12** ^13^C NMR spectrum of FR in CDCl_3_ (125 MHz) 12](#_Toc135380106)

[**Tab. S1** Sequence accession numbers, the corresponding organisms from Fig. 3 and its description 3](#_Toc130315417)

[**Tab. S2** Effect of FR-Core on allocation of *C. elegans* N2 10](#_Toc130315418)

**Fig. S1** Calibration curves with the area under the curve (AUC) for FR (m/z 1002.5) determined by LC/MS: **a** for higher concentrations (0; 0.0001, 0.0005, 0.001, 0.005, 0.01, 0.05, and 0.1 mg/L FR) and **b** for lower concentrations (0, 0.0001, 0.0005, 0.001 mg/mL FR). Equations given in the figures were calculated using Prism (Vers. 9.5.0)

**Fig. S2** FR concentration in n-butanol extracts of C. vaccinii culture in SESOM with chitin (1 g/L) cultivated for 5 days or extracted after inoculation (0 days). The FR concentration was evaluated using HPLC/MS. Three repeats were performed for the control, and four repeats were cultivated for 5 days. The significance was determined using the two-tailed unpaired t-test. P > 0.05 = ns, P < 0.05 = *, P < 0.01 = **, P < 0.001 = ***, P < 0.0001 = ****

**Tab. S1** Sequence accession numbers, corresponding organisms from Fig. 3, and their description

| **Sequence accession No. or Origin** | **Organisms** | **Type and Origin (Clade for Caenorhabditis spp.** (Dayi et al., 2021)**)** |
| --- | --- | --- |
| G5EGU1 (UniProtKB) | *Caenorhabditis elegans* | *Elegans* supergroup |
| B6VBV2 (UniProtKB) | *Caenorhabditis angaria* | *Drosophilae* supergroup, ectophoretic associate of *Metamasius hemipterus* (Sudhaus et al., 2011) |
| A0A261B5L1 (UniProtKB) | *Caenorhabditis latens* | *Elegans* supergroup, strain from Jiangsu and Wuhan City in China (Dey et al., 2012; Félix et al., 2014) |
| Q4VT45 (UniProtKB) | *Caenorhabditis briggsae* | *Elegans* supergroup, entomopathogenic  strain associated with *Serratia* sp (Abebe et al., 2010) |
| A0A2G5VU95 (UniProtKB) | *Caenorhabditis nigoni* | *Elegans* supergroup, isolated in south india and congo from rotting flowers (Kiontke et al., 2011; Félix et al., 2014) |
| E3LXV8 (UniProtKB) | *Caenorhabditis remanei* | *Elegans* supergroup, isolated from isopods obtained from compost heaps (Baird, 1999) |
| A0A1I7UE77 (UniProtKB) | *Caenorhabditis tropicalis* | *Elegans* supergroup, isolated in La Réunion and French Guiana from rotting flowers (Kiontke et al., 2011; Félix et al., 2014) |
| A0A8R1DI86 (UniProtKB) | *Caenorhaditis japonica* | *Elegans* supergroup, associated with *Parastrachia japonensis* (Hironaka et al., 2002) |
| A0A8S1GU46 (UniProtKB) | *Caenorhabditis auriculariae* | Basal group, isolated from fruiting bodies of *Auricularia polytricha* (Tsuda and Futai, 1999) |
| A0A8S1F2E0 (UniProtKB) | *Caenorhabditis bovis* | *Drosophilae* supergroup, associated with *Chrysomya bezziana* (Stevens et al., 2020) |
| KAF8367701.1 (Genbank) | *Pristionchus pacificus* | Satellite model organism associated with scarab beetles (Herrmann et al., 2006) |
| KAI1707081.1 (Genbank) | *Ditylenchus destructor* | Plant parasitic potato root nematode (Zheng et al., 2016) |
| A0A8S9ZE38 (UniProtKB) | *Meloidogyne graminicola* | Plant parasitic root-knot nematodes (Htay et al., 2016) |
| A0A6V7U1D6 (UniProtKB) | *Meloidogyne enterolobii* | Plant parasitic root-knot nematodes (Elling, 2013) |
| KAH7721960.1 (Genbank) | *Aphelenchus avenae* | Fungivorous soil nematode (Kumari, 2012) |
| A0A811JUE8 (UniProtKB) | *Bursaphelenchus okinawaensis* | Associated with longhorn beetle *Monochamus maruokai* (Kanzaki et al., 2008) |
| KAI6178527.1 (Genbank) | *Aphelenchoides besseyi* | Foliar plant parasitic nematode (Jones et al., 2013) |
| WormBase ParaSite - BioProject PRJNA722882: Transcript of Hsc_gene_6303 (Hsc_gene_6303.t1) | *Heterodera schachtii* | Plant pathogenic cyst nematodes (Sijmons, 1993) |
| P50148 (UniProtKB) | *Homo sapiens* | - |

**Fig. S3** IP_1_ accumulation after stimulation without and with 1 mM and 10 µM carbachol (CcH) of HEK293 Gα_q_/Gα_11_-null cells transfected to express only H. schachtii Gα_q_ isoform **a**; H. schachtii Gα_q_ isoform with M3 overexpression **b**; with RIC-8A expression **c**; or with both M3 overexpression and RIC-8A expression **d**. Mean +/- SEM, 3 biological replicates performed in triplicate


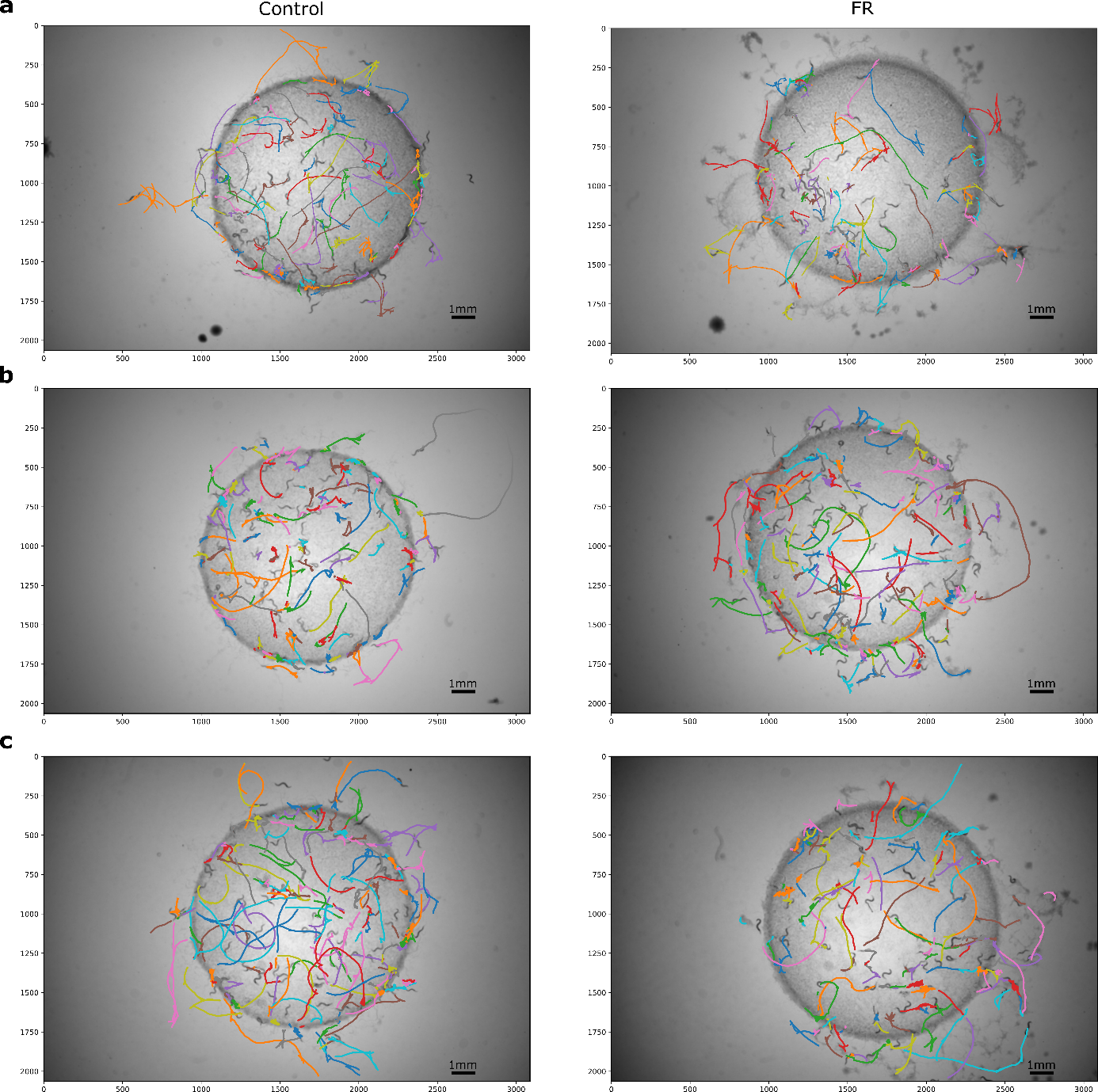


**Fig. S4** Tracking patterns of C. elegans N2 with and without FR. Adult nematodes synchronized using bleach were recorded for 5 minutes on NGM with a Control (1% DMSO mixed with E. coli OP50) or FR (2.5 mM FR/1 % DMSO mixed with E. coli OP50) spot. All experiments were done in three repeats **a**-**c**


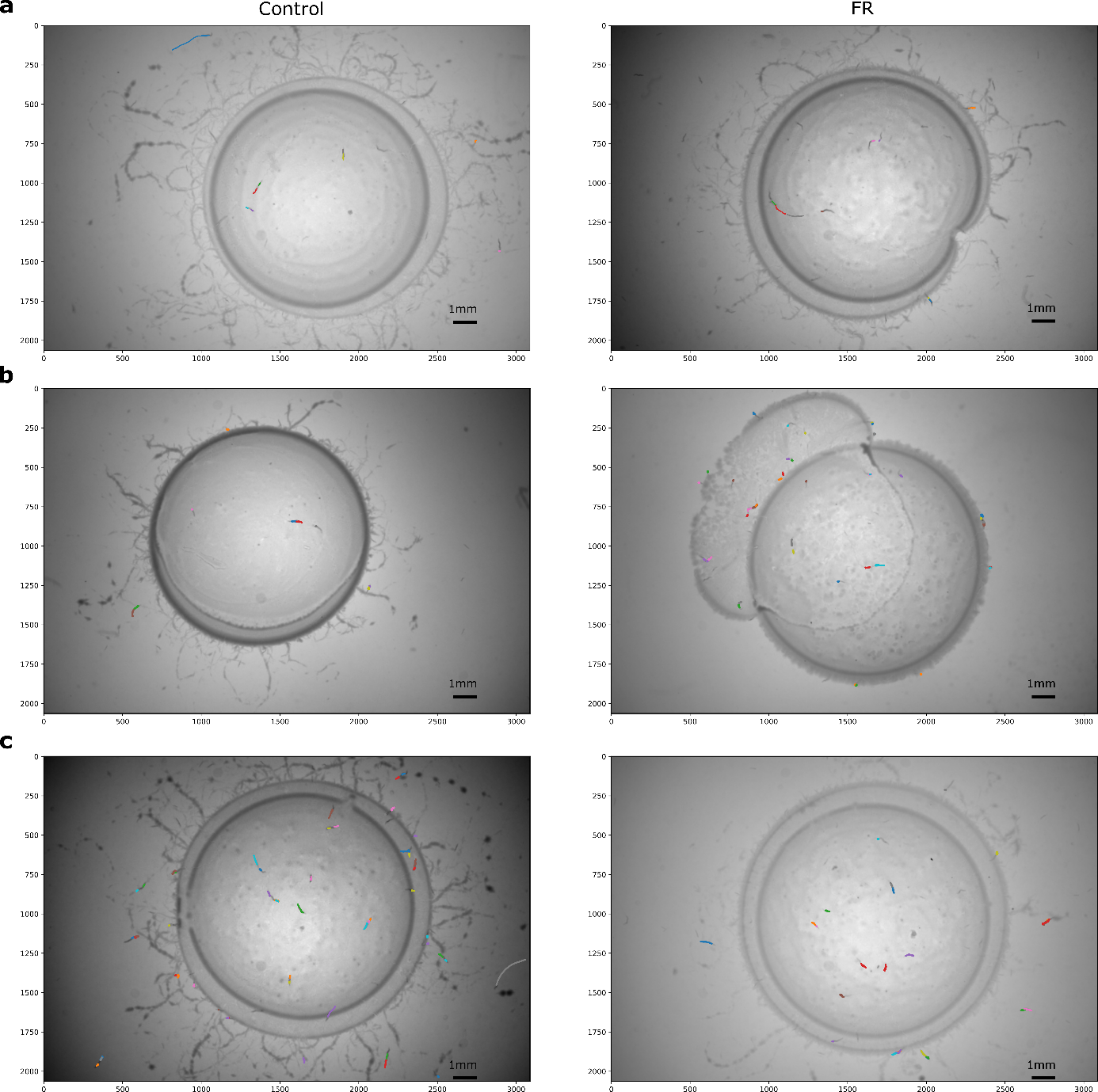


**Fig. S5** Tracking patterns of C. elegans egl-30(ad805) with and without FR. Adult nematodes synchronized using bleach were recorded for 5 minutes on NGM with a Control (1% DMSO mixed with E. coli OP50) or FR (2.5 mM FR/1 % DMSO mixed with E. coli OP50) spot. All experiments were done in three repeats **a**-**c**


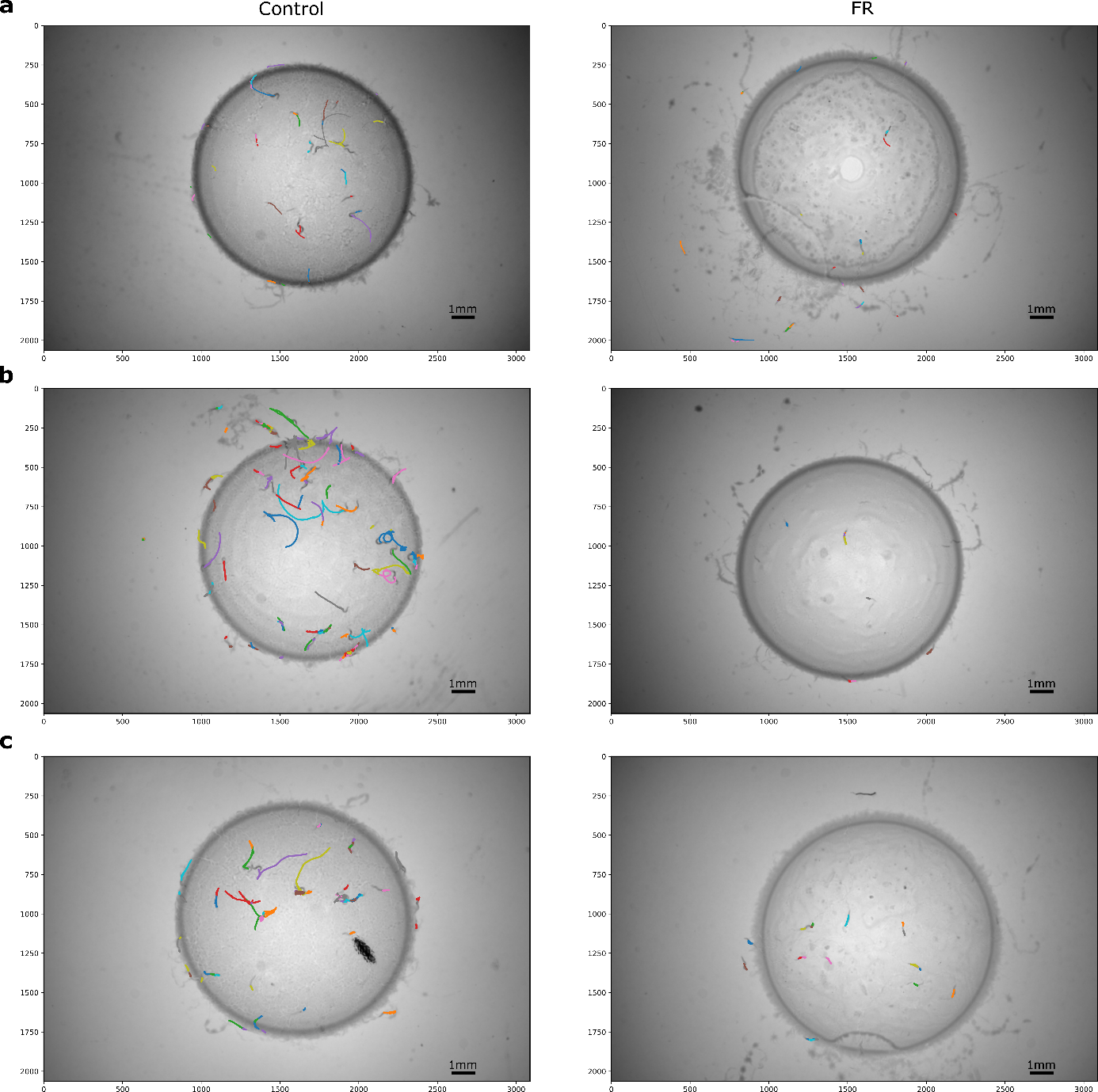


**Fig. S6** Tracking patterns of C. elegans egl-30(n686) with and without FR. Adult nematodes synchronized using bleach were recorded for 5 minutes on NGM with a Control (1% DMSO mixed with E. coli OP50) or FR (2.5 mM FR/1 % DMSO mixed with E. coli OP50) spot. All experiments were done in three repeats **a**-**c**


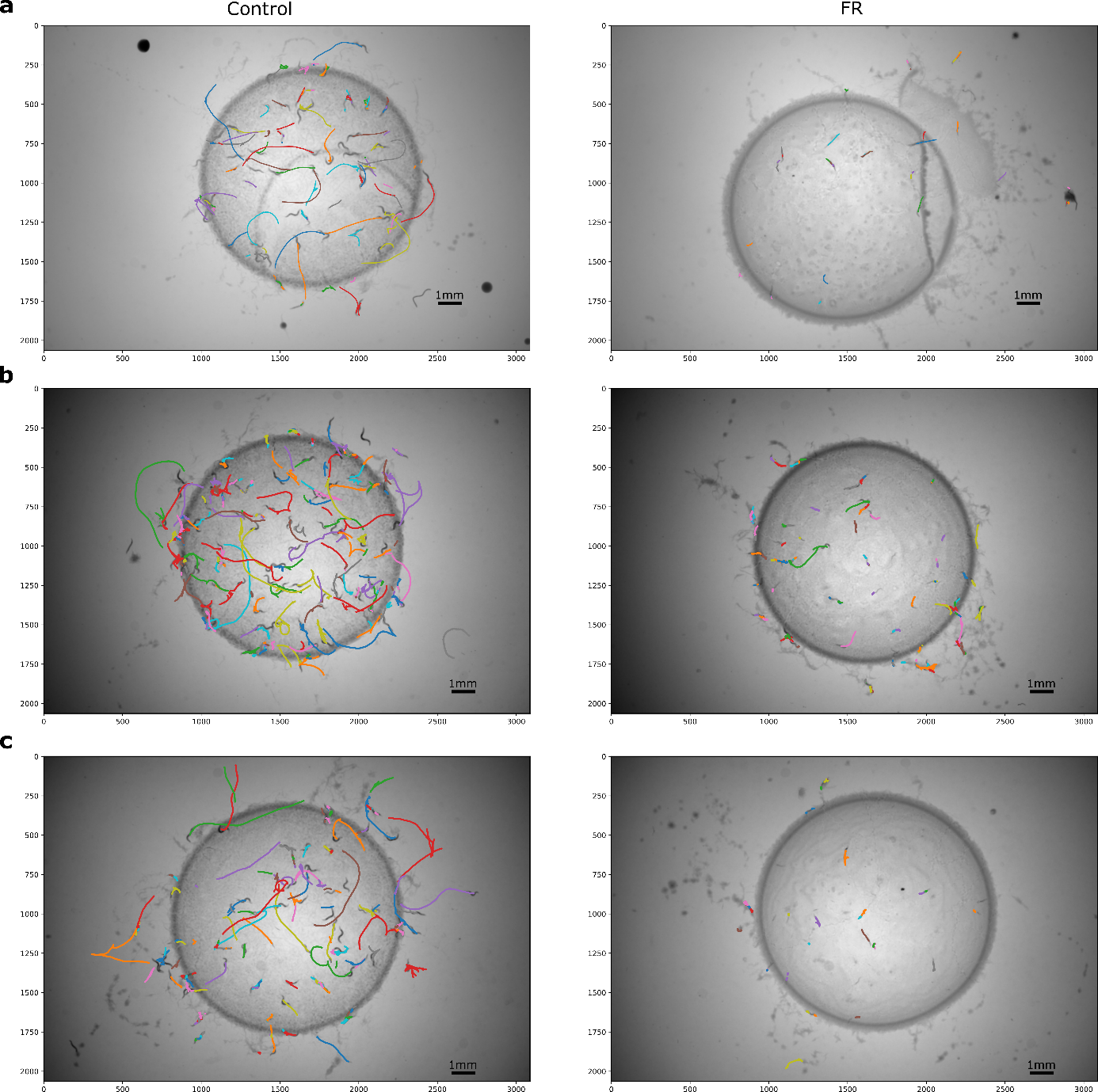


**Fig. S7** Tracking patterns of C. elegans egl-30(ad806) with and without FR. Adult nematodes synchronized using bleach were recorded for 5 minutes on NGM with a Control (1% DMSO mixed with E. coli OP50) or FR (2.5 mM FR/1 % DMSO mixed with E. coli OP50) spot. All experiments were done in three repeats **a**-**c**


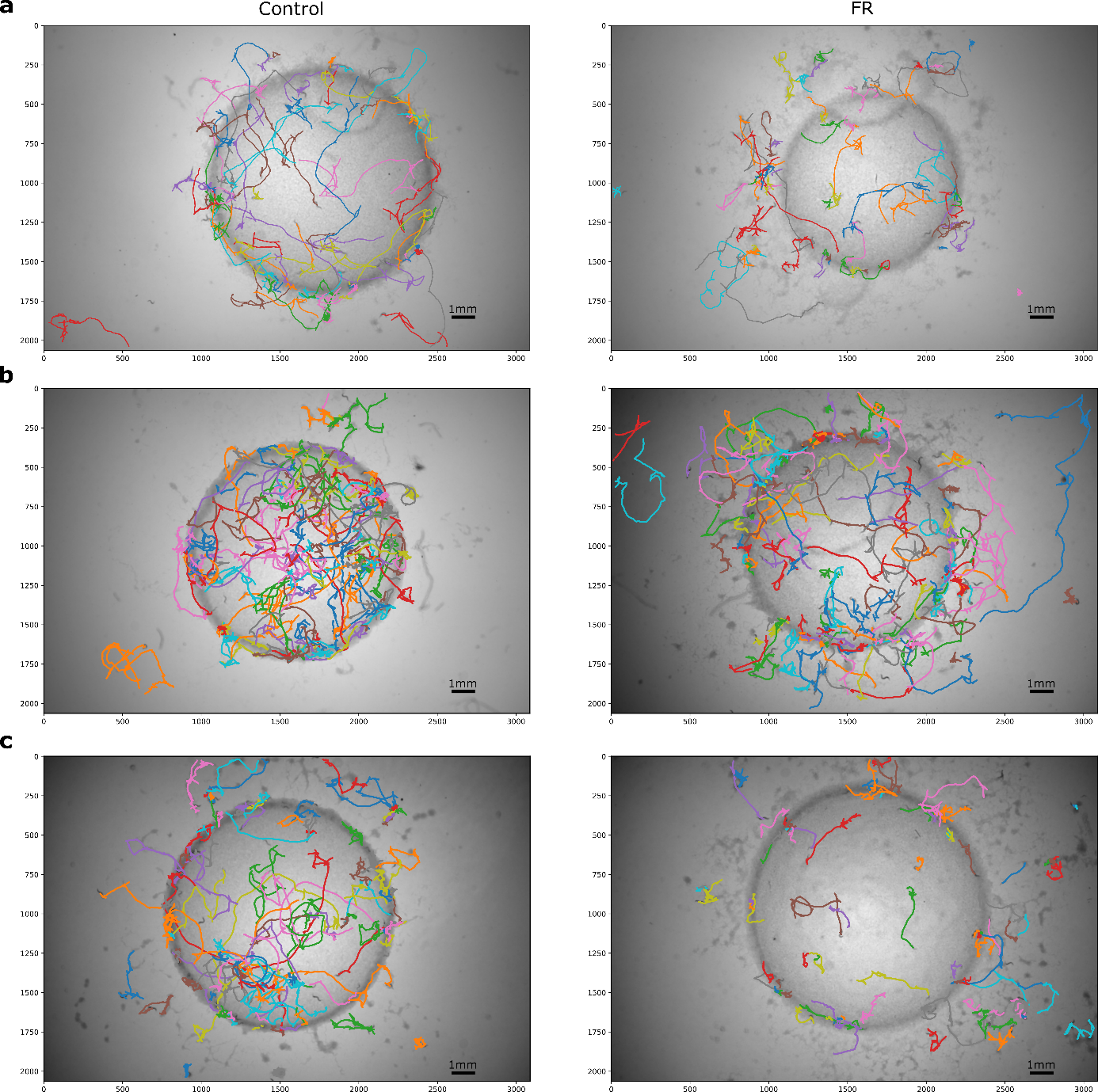


**Fig. S8** Tracking patterns of C. elegans dgk-1(sy428) with and without FR. Adult nematodes synchronized using bleach were recorded for 5 minutes on NGM with a Control (1% DMSO mixed with E. coli OP50) or FR (2.5 mM FR/1 % DMSO mixed with E. coli OP50) spot. All experiments were done in three repeats **a**-**c**


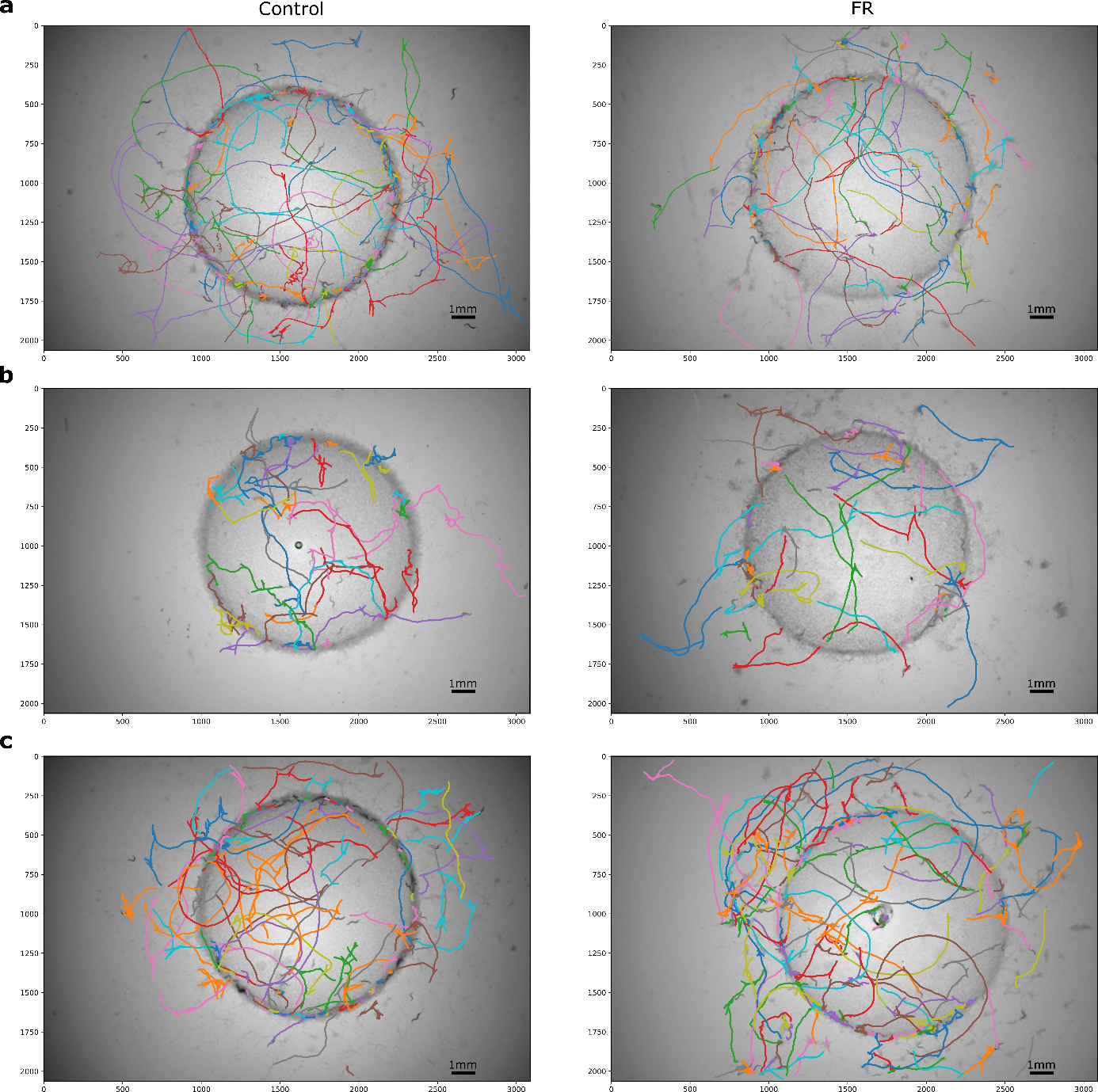


**Fig. S9** Tracking patterns of C. elegans eat-16(sa609) with and without FR. Adult nematodes synchronized using bleach were recorded for 5 minutes on NGM with a Control (1 % DMSO mixed with E. coli OP50) or FR (2.5 mM FR/1 % DMSO mixed with E. coli OP50) spot. All experiments were done in three repeats **a**-**c**

**Tab. S2** Effect of FR-Core on allocation of C. elegans N2. Adult nematodes synchronized using bleach were recorded for 5 minutes on NGM with a Control (1% DMSO mixed with E. coli OP50) or FR-Core (2.5 mM FR-Core/1 % DMSO mixed with E. coli OP50) spot. All experiments were done in three repeats. The allocation was compared at the start and end (5 min) of the video using the modified two sample binomial test (Wong et al., 2014). P > 0.05 = ns, P < 0.05 = *, P < 0.01 = **, P < 0.001 = ***, P < 0.0001 = ****

|  | **Nematodes in lawn (%)** | | | |
| --- | --- | --- | --- | --- |
|  | Control | FR-Core | Modified two  sample binomial  test |  |
| **total** | 92.2 (±4.6) | 94.1 (±4.1) | ns (p=0.3576) |  |


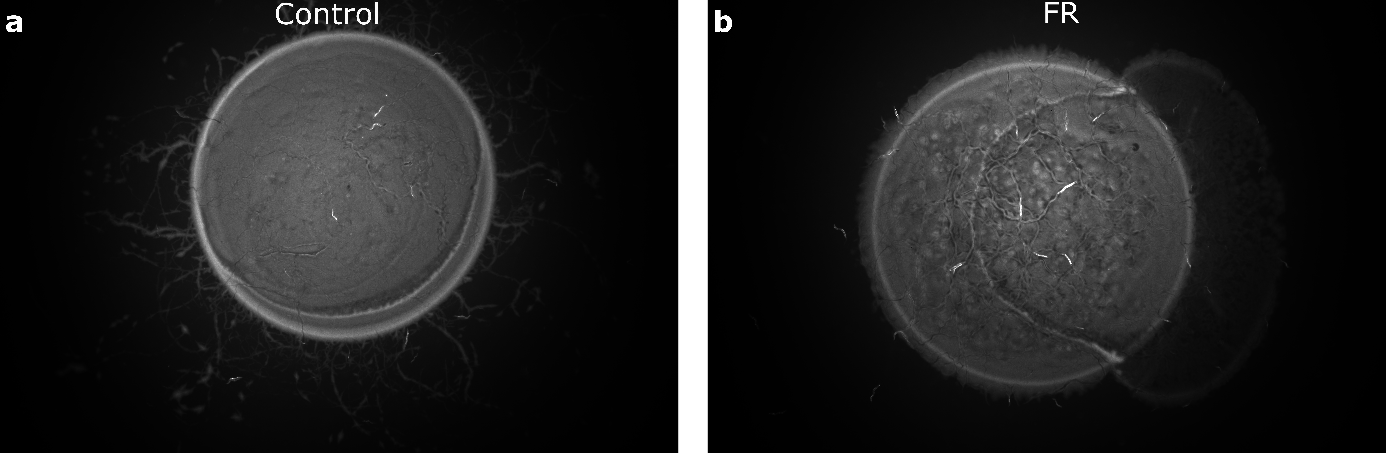


**Fig. S10** Egg-laying adults of C. elegans egl-30(ad805) after 79 hours on plate. Control **a** with 1% DMSO mixed with E. coli OP50. FR **b** with 2.5 mM FR/1% DMSO mixed with E. coli OP50. Images taken in dark field.

**Fig. S11** ^1^H NMR spectrum of FR in CDCl_3_ (600 MHz)

**Fig. S12** ^13^C NMR spectrum of FR in CDCl_3_ (125 MHz)

REFERENCES

Abebe, E., Jumba, M., Bonner, K., and Gray, V., et al. (2010) An entomopathogenic Caenorhabditis briggsae. J Exp Biol 213 Pt 18:3223–3229. doi:10.1242/jeb.043109

Baird, S. E. (1999) Natural and experimental associations of Caenorhabditis remanei with Trachelipus rathkii and other terrestrial isopods. Nematol 1 5:471–475. doi:10.1163/156854199508478

Dayi, M., Kanzaki, N., Sun, S., and Ide, T., et al. (2021) Additional description and genome analyses of Caenorhabditis auriculariae representing the basal lineage of genus Caenorhabditis. Sci Rep 11 1:6720. doi:10.1038/s41598-021-85967-z

Dey, A., Jeon, Y., Wang, G.-X., and Cutter, A. D. (2012) Global population genetic structure of Caenorhabditis remanei reveals incipient speciation. Genetics 191 4:1257–1269. doi:10.1534/genetics.112.140418

Elling, A. A. (2013) Major emerging problems with minor meloidogyne species. Phytopathology 103 11:1092–1102. doi:10.1094/ PHYTO-01-13-0019-RVW

Félix, M.-A., Braendle, C., and Cutter, A. D. (2014) A streamlined system for species diagnosis in Caenorhabditis (Nematoda: Rhabditidae) with name designations for 15 distinct biological species. PLoS One 9 4:e94723. doi:10.1371/journal.pone.0094723

Herrmann, M., Mayer, W. E., and Sommer, R. J. (2006) Nematodes of the genus Pristionchus are closely associated with scarab beetles and the Colorado potato beetle in Western Europe. Zoology (Jena) 109 2:96–108. doi:10.1016/j.zool.2006.03.001

Hironaka, M., Kiontke, K., and Sudhaus, W. (2002) Description of Caenorhabditis japonica n. sp. (Nematoda: Rhabditida) associated with the burrower bug Parastrachia japonensis (Heteroptera: Cydnidae) in Japan. Nematol 4 8:933–941. doi:10.1163/156854102321122557

Htay, C., Peng, H., Huang, W., and Kong, L., et al. (2016) The development and molecular characterization of a rapid detection method for Rice root-knot nematode (Meloidogyne graminicola). Eur J Plant Pathol 146 2:281–291. doi:10.1007/s10658-016-0913-y

Jones, J. T., Haegeman, A., Danchin, E. G. J., and Gaur, H. S., et al. (2013) Top 10 plant-parasitic nematodes in molecular plant pathology. Mol Plant Pathol 14 9:946–961. doi:10.1111/mpp.12057

Kanzaki, N., Maehara, N., Aikawa, T., and Togashi, K. (2008) First report of parthenogenesis in the genus Bursaphelenchus Fuchs, 1937: a description of Bursaphelenchus okinawaensis sp. nov. isolated from Monochamus maruokai (Coleoptera: Cerambycidae). Zoolog Sci 25 8:861–873. doi:10.2108/zsj.25.861

Kiontke, K. C., Félix, M.-A., Ailion, M., and Rockman, M. V., et al. (2011) A phylogeny and molecular barcodes for Caenorhabditis, with numerous new species from rotting fruits. BMC Evol Biol 11:339. doi:10.1186/1471-2148-11-339

Kumari, S. (2012) Aphelenchus avenae (Nematoda: Aphelenchidae) under the rhizosphere of Brassica napus. Helminthologia 49 1:57–59. doi:10.2478/s11687-012-0009-y

Sijmons, P. C. (1993) Plant-nematode interactions. Plant Mol Biol 23 5:917–931. doi:10.1007/BF00021809

Stevens, L., Rooke, S., Falzon, L. C., and Machuka, E. M., et al. (2020) The Genome of Caenorhabditis bovis. Curr Biol 30 6:1023-1031.e4. doi:10.1016/j.cub.2020.01.074

Sudhaus, W., Giblin-Davis, R., and Kiontke, K. (2011) Description of Caenorhabditis angaria n. sp. (Nematoda: Rhabditidae), an associate of sugarcane and palm weevils (Coleoptera: Curculionidae). Nematol 13 1:61–78. doi:10.1163/138855410X500334

Tsuda, K. and Futai, K. (1999) Description of Caenorhabditis auriculariae n. sp.(Nematoda: Rhabditida) from Fruiting Bodies of Auricularia polytricha. Jpn. J. Nematol. 29 1:18–23. doi:10.3725/jjn1993.29.1_18

Wong, K.-F., Wong, W.-K., and Lin, M.-S. (2014) Forward selection two sample binomial test. J Data Sci 12 4:279–294

Zheng, J., Peng, D., Chen, L., and Liu, H., et al. (2016) The Ditylenchus destructor genome provides new insights into the evolution of plant parasitic nematodes. Proc Biol Sci 283 1835. doi:10.1098/rspb.2016.0942
